# Supplementary material for: Baseline and acquired resistance to bedaquiline, linezolid and pretomanid, and impact on treatment outcomes in four tuberculosis clinical trials containing pretomanid
Source: PLOS Glob Public Health. 2023 Oct 18;3(10):e0002283. doi: 10.1371/journal.pgph.0002283 (PMC10584172; doi:10.1371/journal.pgph.0002283)
Supplement: S1 Text — (DOCX) [file pgph.0002283.s001.docx]

**S1 Text. Supplementary methods and references**

**Methods**

1. List of Ethics Committees and/or Institutional Review Boards who granted approval for the 4 trial(s).

| **Country** | **Site number** | | | | **Approving institution** |
| --- | --- | --- | --- | --- | --- |
|  | **STAND** | **Nix-TB** | **ZeNix** | **SimpliciTB** |  |
| Malaysia | 0201 |  |  |  | Medical Research Ethics Committee, Ministry of Health Malaysia |
| Malaysia | 0203 |  |  |  | Universiti Kebangsaan Malaysia (UKM) Research and Ethics Committee |
| Malaysia | 0204 |  |  |  | Research Management Institute (RMI), Universiti Teknologi MARA (UiTM) |
| Malaysia |  |  |  | 1601 | Medical Research Ethics Committee Institute for Health Management (Central Ethics) |
| South Africa | 0301 |  |  | 1004 | University of Cape Town, Faculty of Health Sciences Human Research Ethics Committee (Local Ethics) |
| South Africa | 0302, 0303  0304, 0305  0307, 0308  0309, 0310  0311, 0312  0313 | 001, 004 | 1001, 1003 1004, 1011 | 1001, 1002, 1003, 1005, 1006, 1007, 1008, 1009, 1010, 1011 | University of Witwatersrand Human Research Ethics Committee (Medical) (Central Ethics) |
| South Africa |  | 002 |  |  | Pharma-Ethics |
| Tanzania | 0401 |  |  |  | - Mbeya Medical Research and Ethics Committee, Mbeya Consultant Hospital - National Institute of Medical Research |
| Tanzania |  |  |  | 1401 | Mbeya Medical Research and Ethics Committee (Local Ethics) |
| Tanzania | 0402 |  |  |  | - Ifakara Health Institute Institutional Review Board - National Institute of Medical Research |
| Tanzania |  |  |  | 1402 | Ifakara Health Institute Institutional Review Board (Local Ethics) |
| Tanzania |  |  |  | 1401, 1402, 1403, 1404 | National Health Research Ethics Review Committee (Central Ethics) |
| Tanzania |  |  |  | 1404 | Kilimanjaro Christian Medical College Research Ethics and Review Committee (CRERC) (Local Ethics) |
| Thailand | 0501 |  |  |  | - Central Research Ethics Committee (CREC) Central Ethics Committee - Ethical Review Committee of Central Chest Institute of Thailand Local Ethics Committee - Institute Review Board of Bamrasnaradura Institute Infectious Diseases Institute Local Ethics Committee |
| Ukraine | 0601 |  |  |  | LEC Mun. Tuberculosis Hospital #1 Local Ethics Committee |
| Ukraine | 0602 |  |  |  | LEC SI National Institute of Phthisiology and Pulmonology  named F.G.Yanovskyy of AMS of Ukraine Local Ethics Committee |
| Kenya | 0701, 0702 |  |  |  | Kenya Medical Research Institute Scientific Ethics Review Unit (KEMRI SERU) |
| Philippines | 1002 |  |  |  | University of the Philippines Manila Research Ethics Board Local Ethics Committee |
| Philippines | 1003 |  |  |  | - Lung Center of The Philippines Ethics Committee (Local Ethics) - Lung Center of the Philippines Technical Review Board (Local Ethics) |
| Philippines | 1004 |  |  |  | Tropical Disease Foundation Institutional Review Board Local Ethics Committee |
| Philippines |  |  |  | 1701 | Makati Medical Center Institutional Review Board (Local Ethics) |
| Philippines |  |  |  | 1702 | Ethics Review Committee of the Lung Center of Philippines (Local Ethics) |
| Philippines |  |  |  | 1703 | Institutional Review Board - Jose R. Reyes Memorial Medical Center (Local Ethics) |
| Uganda | 1301 |  |  |  | - University of Makerere Scientific and Biomedical Studies Institutional Review Board - Uganda National Council for Science and Technology (UNCST) |
| Uganda |  |  |  | 1501 | - Uganda National Council for Science and Technology (UNCST) (Central Ethics) - Makerere University College of Health Sciences, School of Biomedical Sciences (Local Ethics) |
| Georgia | 1501 |  |  |  | LEC of National Center for Tuberculosis and Lung Diseases (Local Ethics) |
| Georgia |  |  | 1105 | 1105 | IEC of National Center for Tuberculosis and Lung Disease (Local Ethics) |
| Russia |  |  | 1206 | 1206 | Local Ethics Committee at Central Research Institute of Tuberculosis, Moscow |
| Russia |  |  | 1207 |  | National Medical Research Centre of Phthisiopulmonology and Infectious Diseases, Yekaterinburg (Local Ethics) |
| Russia |  |  |  | 1207 | - Ethics Committee at Ural Scientific Research Institution of Phthiziopulmonology (Local Ethics) - Ethics Committee at Ural State Medical University (Local Ethics) |
| Russia |  |  | 1208 | 1208 | Ethics Committee at FSBI “St. Petersburg Research Institute of  Phthisiopulmonology (Local Ethics) |
| Russia |  |  | 1209 | 1209 | Ethics Committee at Ural Scientific Research Institution of  Phthiziopulmonology (Local Ethics) Yekaterinburg |
| Russia |  |  | 1210 | 1210 | - Moscow City Independent Ethics Committee (Local Ethics)  - The Russian Federation Ministry of Health, Department of State Regulation of Circulation of Medicines, Ethics Council (Central Ethics) |
| Russia |  |  |  | 1206, 1207, 1208, 1209 | The Russian Federation Ministry of Health, Department of State Regulation of Circulation of Medicines, Ethics Council (Central Ethics) |
| Moldova |  |  | 2201 |  | The National Committee for Ethical Review of Clinical Trials (Central Ethics) |
| Brazil |  |  |  | 2001, 2002 | Comissão Nacional de Ética em Pesquisa (CONEP) (Central Ethics) |
| Brazil |  |  |  | 2001 | Comitê de Ética em Pesquisa do Instituto Nacional de Infectologia Evandro Chagas - INI – Fiocruz (Local Ethics) |
| Brazil |  |  |  | 2002 | Comitê de Ética em Pesquisa da Escola Nacional de Saúde Pública Sérgio Arouca – ENSP (Local Ethics) |

1. Schedule of sputum cultures across trials

| **Period** | **Screen-ing** | **Treatment** | | | | | | | | | | | | | | | | **Follow-Up** | | | | | | | | | | |
| --- | --- | --- | --- | --- | --- | --- | --- | --- | --- | --- | --- | --- | --- | --- | --- | --- | --- | --- | --- | --- | --- | --- | --- | --- | --- | --- | --- | --- |
| **Visit^1,2^** | D-14/D-9 to D-1 | D1 (Baseline) | W1 | W2 | W3 | W 4 | W5 | W6 | W7 | W8 (M2) | W10 | W12 (M3) | W16/17 (M4)^4^ | W20 | W22/23 (M5) | W26 (M6) ^5^ | FUW4 (FUM1) | | FUW8 (FUM2) | FUW12/13 (FUM3) | FUW26 (FUM6) | FUW39 (FUM9) | FUW52 (FUM12) | FUW65 (FUM15) | FUW78 (FUM18) | FUM21 | FUM24 |  |
|  |  |  |  |  |  |  |  |  |  |  |  |  |  |  |  |  |  |  |  |  |  |  |  |  |  |  |  |  |
| **STAND** | X | X | X | X | X | X | X | X | X | X |  | X | X |  | X | X |  | |  | X | X | X | X |  | X |  |  |  |
| **Nix-TB^3^** | X | X | X | X |  | X |  | X |  | X |  | X | X | X |  | X | X | | X | X | X | X | X | X | X | X | X |  |
| **ZeNix^3^** | X | X | X | X | X | X |  | X |  | X | X | X | X | X | X | X | X | | X | X | X | X | X | X | X |  |  |  |
| **SimpliciTB** | X | X | X | X | X | X | X | X | X | X |  | X | X |  | X | X |  | |  | X | X | X | X |  | X |  |  |  |

^1^D = day, FU = follow-up post end-of-treatment, M = month, W = week

^2^Additional unscheduled or early withdrawal visits could take place at any point throughout the trials.

^3^Nix-TB and ZeNix participants with a sputum culture positive for MTB at W16 could have their treatment extended to 9 months. In this case, additional sputum cultures were to be performed at W30, W34 and W39.

^4^STAND and SimpliciTB included a 4-month treatment arm, in addition to a 6-month treatment arm.

1. REMA MIC assay

Pretomanid stock solutions were prepared in DMSO, and further diluted in 7H9 media (supplemented with 10% OADC/4% glycerol/1% Tween) to create a working solution immediately prior to use. Two-fold serial dilutions in 100 µl 7H9 (See Table S2) were performed in the 96-well plates (1.25% DMSO final concentration in the top drug concentration). Two drug-free wells were included as the growth control (GC) for each isolate. Plates were inoculated with 100 µl bacterial suspension prepared from a MGIT seed culture, used 1-5 days after flagging positive, as per MGIT MIC inoculum preparation guidelines [1]. Plates were sealed in plastic bags and incubated for 7 days at 37°C, after which 20 µl 0.01% resazurin dye (prepared fresh from 0.1% frozen stocks) was added to each well and re-incubated at 37°C, protected from light. Plates were observed after 24 and 48 hours for a color change in GC wells, after which drug-containing wells were assessed and the MIC determined as the lowest concentration of drug that prevented this color change.

1. WGS analyses

Sequence reads were mapped to the H37Rv reference genome (RefSeq accession: NC_000962.3) using bwa mem v.0.7.17 [2], and alignments sorted using samtools v.1.12 [3]. Site Statistics were generated using bcftools mpileup v.1.12 and gene annotation generated using snpEff (v.4.3.1t) software [4]. Only genome sequences showing mean read depth of at least 20x were included in the analyses, with 88% of baseline samples having mean read depth of >100x. Sequence purity (% MTB complex) was determined using Kraken [5], to exclude potential contamination with non-tuberculous mycobacteria sequence. See Table S3 for the full list of known pretomanid and bedaquiline resistance conferring genes, genome positions and upstream regions that were analyzed for variants. Linezolid-resistant conferring variants were determined using TB-profiler v.4.1.1 and database tbdb_a9fac19_Feb 16 2022) [6], which called resistance based any of the following variants: *rplC*: Cys154Arg; *rrl*: 2299G>T; *rrl*: 2814G>T. In isolates with phenotypic linezolid resistance, any other variants in *rplC* and *rrl* were also reviewed from the TB-profiler outputs. The percentage of variants of interest, as determined by the in-house bioinformatics pipeline and/or the TB-profiler, in the genes conferring resistance to pretomanid, bedaquiline and linezolid were verified by viewing the BAM files against the H37Rv genome using the genome viewer IGV v.2.15.2. The percentage of variants was calculated from the proportion of sequence reads showing variants of interest in relation to the sequence depth at the corresponding genomic position. Closely located low proportions SNPs were investigated to find out whether they were on different reads which may indicate various subpopulations. Also in the genome viewer, we looked for signature of insertion sequences in the genes of interest [7] in the isolates with undetermined genetic mechanism of resistance. Maximum likelihood phylogenetic trees using IQ-TREE (v2.0.3) with a General Time Reversible model of nucleotide substitution (model selection restricted to those supported by RAxML); branch support values were determined using 1000 bootstrap replicates) [8]. Phylogenetic comparisons for the tree construction filter out all mixed sites and INDELS, but these sites were included in pairwise comparison of vcf files to look for variants that may be associated with acquired resistance.

**References**

1. World Health Organization. Technical manual for drug susceptibility testing of medicines used in the treatment of tuberculosis [WHO/CDS/TB/2018.24]<https://apps.who.int/iris/handle/10665/275469> (accessed 12 Aug 2021). 2018.

2. Li H. Aligning sequence reads, clone sequences and assembly contigs with BWA-MEM. arXiv preprint arXiv:13033997. 2013.

3. Li H, Handsaker B, Wysoker A, Fennell T, Ruan J, Homer N, et al. The Sequence Alignment/Map format and SAMtools. Bioinformatics. 2009;25(16):2078-9. Epub 2009/06/10. doi: 10.1093/bioinformatics/btp352. PubMed PMID: 19505943; PubMed Central PMCID: PMCPMC2723002.

4. Cingolani P, Platts A, Wang le L, Coon M, Nguyen T, Wang L, et al. A program for annotating and predicting the effects of single nucleotide polymorphisms, SnpEff: SNPs in the genome of Drosophila melanogaster strain w1118; iso-2; iso-3. Fly (Austin). 2012;6(2):80-92. Epub 2012/06/26. doi: 10.4161/fly.19695. PubMed PMID: 22728672; PubMed Central PMCID: PMCPMC3679285.

5. Wood DE, Salzberg SL. Kraken: ultrafast metagenomic sequence classification using exact alignments. Genome Biol. 2014;15(3):R46. Epub 20140303. doi: 10.1186/gb-2014-15-3-r46. PubMed PMID: 24580807; PubMed Central PMCID: PMCPMC4053813.

6. Phelan JE, O'Sullivan DM, Machado D, Ramos J, Oppong YEA, Campino S, et al. Integrating informatics tools and portable sequencing technology for rapid detection of resistance to anti-tuberculous drugs. Genome Med. 2019;11(1):41. Epub 20190624. doi: 10.1186/s13073-019-0650-x. PubMed PMID: 31234910; PubMed Central PMCID: PMCPMC6591855.

7. Antoine R, Gaudin C, Hartkoorn RC. Intragenic Distribution of IS6110 in Clinical Mycobacterium tuberculosis Strains: Bioinformatic Evidence for Gene Disruption Leading to Underdiagnosed Antibiotic Resistance. Microbiol Spectr. 2021;9(1):e0001921. Epub 2021/07/22. doi: 10.1128/Spectrum.00019-21. PubMed PMID: 34287057; PubMed Central PMCID: PMCPMC8552512.

8. Witney AA, Bateson AL, Jindani A, Phillips PP, Coleman D, Stoker NG, et al. Use of whole-genome sequencing to distinguish relapse from reinfection in a completed tuberculosis clinical trial. BMC Med. 2017;15(1):71. Epub 2017/03/30. doi: 10.1186/s12916-017-0834-4. PubMed PMID: 28351427; PubMed Central PMCID: PMCPMC5371199.

9. Cortes T, Schubert OT, Rose G, Arnvig KB, Comas I, Aebersold R, et al. Genome-wide mapping of transcriptional start sites defines an extensive leaderless transcriptome in Mycobacterium tuberculosis. Cell Rep. 2013;5(4):1121-31. Epub 20131121. doi: 10.1016/j.celrep.2013.10.031. PubMed PMID: 24268774; PubMed Central PMCID: PMCPMC3898074.

10. <http://galaganlab.bu.edu/tbdb_sysbio/operon/Rv3504.html> [06 Mar 2023].

11. Radhakrishnan A, Kumar N, Wright CC, Chou TH, Tringides ML, Bolla JR, et al. Crystal structure of the transcriptional regulator Rv0678 of Mycobacterium tuberculosis. J Biol Chem. 2014;289(23):16526-40. Epub 20140415. doi: 10.1074/jbc.M113.538959. PubMed PMID: 24737322; PubMed Central PMCID: PMCPMC4047419.

12. Sala C, Haouz A, Saul FA, Miras I, Rosenkrands I, Alzari PM, et al. Genome-wide regulon and crystal structure of BlaI (Rv1846c) from Mycobacterium tuberculosis. Mol Microbiol. 2009;71(5):1102-16. Epub 20090116. doi: 10.1111/j.1365-2958.2008.06583.x. PubMed PMID: 19154333.

13. Roback P, Beard J, Baumann D, Gille C, Henry K, Krohn S, et al. A predicted operon map for Mycobacterium tuberculosis. Nucleic Acids Res. 2007;35(15):5085-95. Epub 20070725. doi: 10.1093/nar/gkm518. PubMed PMID: 17652327; PubMed Central PMCID: PMCPMC1976454.

14. Sassetti CM, Rubin EJ. Genetic requirements for mycobacterial survival during infection. Proc Natl Acad Sci U S A. 2003;100(22):12989-94. Epub 20031020. doi: 10.1073/pnas.2134250100. PubMed PMID: 14569030; PubMed Central PMCID: PMCPMC240732.

15. Rengarajan J, Bloom BR, Rubin EJ. Genome-wide requirements for Mycobacterium tuberculosis adaptation and survival in macrophages. Proc Natl Acad Sci U S A. 2005;102(23):8327-32. Epub 20050531. doi: 10.1073/pnas.0503272102. PubMed PMID: 15928073; PubMed Central PMCID: PMCPMC1142121.

16. Griffin JE, Gawronski JD, Dejesus MA, Ioerger TR, Akerley BJ, Sassetti CM. High-resolution phenotypic profiling defines genes essential for mycobacterial growth and cholesterol catabolism. PLoS Pathog. 2011;7(9):e1002251. Epub 20110929. doi: 10.1371/journal.ppat.1002251. PubMed PMID: 21980284; PubMed Central PMCID: PMCPMC3182942.

17. Pawelczyk J, Brzostek A, Minias A, Plocinski P, Rumijowska-Galewicz A, Strapagiel D, et al. Cholesterol-dependent transcriptome remodeling reveals new insight into the contribution of cholesterol to Mycobacterium tuberculosis pathogenesis. Sci Rep. 2021;11(1):12396. Epub 20210611. doi: 10.1038/s41598-021-91812-0. PubMed PMID: 34117327; PubMed Central PMCID: PMCPMC8196197.
