# Supplementary material for: Baseline and acquired resistance to bedaquiline, linezolid and pretomanid, and impact on treatment outcomes in four tuberculosis clinical trials containing pretomanid
Source: PLOS Glob Public Health. 2023 Oct 18;3(10):e0002283. doi: 10.1371/journal.pgph.0002283 (PMC10584172; doi:10.1371/journal.pgph.0002283)
Supplement: S1 Table — (DOCX) [file pgph.0002283.s003.docx]

**S1 Table: Disposition of baseline MTB isolates from the 4 trials.**

| **Participant category** | | **STAND** | **Nix-TB** | **ZeNiX** | **Simplici-TB** | **Total** |
| --- | --- | --- | --- | --- | --- | --- |
| Enrolled | | 284 | 109 | 181 | 455 | 1029 |
| Without positive primary culture from Screening to Week 4 | | 0 | 16 | 41 | 3 | 60 |
| With alternative isolate from pre-screening | | 0 | 0 | 3 | 0 | 3 |
| Without baseline isolates for further characterization for other reasons (e.g. contamination, viability issues, incorrect treatment assignment) | | 72 | 35 | 0 | 5 | 112 |
| With phenotypic DST (MIC/DST in the MGIT or REMA) at baseline | Pretomanid | 212 | 57 | 143 | 447 | 859 |
|  | Bedaquiline | *NA* | 58 | 143 | 447 | 648 |
|  | Linezolid | *NA* | 58 | 143 | *NA* | 201 |
| With WGS (genotypic DST) | | 48 | 56 | 143 | 447 | 694 |
| With both phenotypic and genotypic data | Pretomanid | 45 | 56 | 143 | 447 | 691 |
|  | Bedaquiline | *NA* | 56 | 143 | 447 | 646 |
|  | Linezolid | *NA* | 56 | 143 | *NA* | 199 |

*NA* = not applicable
