# Supplementary material for: Baseline and acquired resistance to bedaquiline, linezolid and pretomanid, and impact on treatment outcomes in four tuberculosis clinical trials containing pretomanid
Source: PLOS Glob Public Health. 2023 Oct 18;3(10):e0002283. doi: 10.1371/journal.pgph.0002283 (PMC10584172; doi:10.1371/journal.pgph.0002283)
Supplement: S2 Table — (DOCX) [file pgph.0002283.s004.docx]

**S2 Table: Drug concentrations tested in the MIC and DST analyses.**

| **Drug** | **Method** | **Isolate range tested, H37Rv acceptable range or critical concentration (mg/L)** |
| --- | --- | --- |
| **MIC** | | |
| Bedaquiline | MGIT | Isolates: 0.125-1 |
|  |  | H37Rv: 0.125-0.5 |
| Linezolid | MGIT | Isolates: 0.25-1 |
|  |  | H37Rv: 0.5-1 |
| Pretomanid | MGIT | Isolates: 0.03-1 |
|  |  | H37Rv: 0.06-0.25 |
|  | REMA | Isolates: 0.03-4 |
|  |  | H37Rv: 0.06-0.25 |
| Moxifloxacin* | REMA | Isolates: 0.06-8 |
|  |  | H37Rv: 0.06-0.25 |
| **DST** | | |
| Streptomycin* | MGIT | 1 |
| Isoniazid* | MGIT | 0.1 |
| Rifampicin* | MGIT | 1 |
| Ethambutol* | MGIT | 5 |
| Moxifloxacin* | MGIT | 0.25 (ZeNix and SimpliciTB); 0.5 (Nix), 2 (STAND) |
| Pyrazinamide* | MGIT | 100 |
| Kanamycin* | MGIT | 2.5 |

*All lyophilized drug powders from Beckon & Dickinson
