# Supplementary material for: Baseline and acquired resistance to bedaquiline, linezolid and pretomanid, and impact on treatment outcomes in four tuberculosis clinical trials containing pretomanid
Source: PLOS Glob Public Health. 2023 Oct 18;3(10):e0002283. doi: 10.1371/journal.pgph.0002283 (PMC10584172; doi:10.1371/journal.pgph.0002283)
Supplement: S3 Table — (DOCX) [file pgph.0002283.s005.docx]

**S3 Table. Genes and upstream regions screened for potential resistance associated variants for pretomanid and bedaquiline.**

| **Rv Number** | **Synonym** | **Strand** | **Leaderless^[9]^** | **UTR**  **Category^[9],a^** | **5'UTR length (bp)^[9]^** | **Co-expressed^[10]^** | **Genomic co-ordinates H37Rv_(NC_000962.3)** | | **Selected upstream region** | | **Justification** |  |
| --- | --- | --- | --- | --- | --- | --- | --- | --- | --- | --- | --- | --- |
|  |  |  |  |  |  |  | **Start** | **End** | **Start** | **End** |  | |
| **Pretomanid Resistance genes** | | | | | | | | | | | |  |
| Rv0407 | *fgd1* | + | Y |  | 0 | N | 490783 | 491793 | 490683 | 490782 | leaderless, 100 bp upstream | |
| Rv1173 | *fbiC* | + | N | SD | 76 | N | 1302931 | 1305501 | 1302755 | 1302930 | 176 bp upstream = 76 bp UTR+ 100 bp | |
| Rv2983 | *fbiD* | + | N |  |  | N | 3339118 | 3339762 | 3339000 | 3339117 | 118 bp upstream; the whole intergenic region | |
| Rv3261 | *fbiA* | + | Y |  | 0 | N | 3640543 | 3641538 | 3640443 | 3640542 | leaderless, 100 bp upstream | |
| Rv3262 | *fbiB* | + | N |  |  | N | 3641535 | 3642881 |  |  |  | |
| Rv3547 | *ddn* | + | Y |  | 0 | N | 3986844 | 3987299 | 3986733 | 3986843 | 111 bp upstream; the whole intergenic region | |
| Rv1854c | *ndh* | - | N | SD | 45 | N | 2101651 | 2103042 | 2103043 | 2103187 | 145 bp upstream =45 bp UTR + 100 bp | |
| **Bedaquiline Resistance genes** | | | | | | | | | | | |  |
| Rv0678 | *mmpR5* | + | Y |  |  | Y | 778990 | 779487 | 778906 | 778989 | 84 bp the whole intergenic region+ the 2 previous genes Rv0677c and Rv0676c ^[11]^ | |
| Rv0677c | *mmpS5* |  | N |  |  |  | 778481 | 778905 |  |  | strong positive correlation (r=0.86623) with Rv0678 | |
| Rv0676c | *mmpL5* | - | N | U | 120 |  | 775586 | 778480 |  |  | strong positive correlation (r=0.80825) with Rv0678 | |
| Rv1304 | *atpB* | + | N |  |  | Y^[12][13]^ | 1460244 | 1460996 | 1459510 | 1460243 | the whole operon starting with Rv1303 (1459510-1460243) | |
| Rv1305 | *atpE* | + | Y |  |  |  | 1461045 | 1461290 | 1460997 | 1461044 | 48 bp upstream; the whole intergenic region | |
| Rv2535c | *pepQ* | - | Y |  |  | N | 2859300 | 2860418 | 2860419 | 2860518 | 101 bp upstream= 1 bp UTR + 100 bp | |
| Rv1979c | *Rv1979c* | - | N | U | 419 | N | 2221719 | 2223164 | 2223165 | 2223683 | 1. upstream= 419 bp UTR + 100 bp | |

^a^Criteria for selecting the up-stream regions:

- 100 bp upstream of the un-translated region (UTR) sequence, if any, as determined by [9] is a conservative approach to capture the motif.

The whole operon whenever there is evidence that the gene exists in an operon or there is a strong correlation (r>0.6) between expression of the gene products [10].

- UTR category was defined for genes with a primary Transcription Start Site (TSS) mapped more than 5 bp from the annotated translation start site. SD: Shine-Dalgarno; U/UTR: Un-translated region; L: Leaderless. Data from [9].
