# Supplementary material for: Baseline and acquired resistance to bedaquiline, linezolid and pretomanid, and impact on treatment outcomes in four tuberculosis clinical trials containing pretomanid
Source: PLOS Glob Public Health. 2023 Oct 18;3(10):e0002283. doi: 10.1371/journal.pgph.0002283 (PMC10584172; doi:10.1371/journal.pgph.0002283)
Supplement: S4 Table — (DOCX) [file pgph.0002283.s006.docx]

**S4 Table: Participants who were exposed to clofazimine for >2 weeks prior to enrolment and did not show bedaquiline baseline resistance in Nix-TB and ZeNix trials.**

| **Study** | **Alternative ID** | **Duration of exposure (days)** |
| --- | --- | --- |
| Nix-TB | NX002 | 47 |
|  | NX003 | 228 |
|  | NX004 | 227 |
|  | NX005 | 27 |
|  | NX006 | 297 |
|  | NX007 | 60 |
|  | NX008 | 81 |
|  | NX009 | 20 |
|  | NX010 | 22 |
|  | NX011 | 18 |
|  | NX014 | 1865 |
|  | NX015 | 36 |
|  | NX016 | 16 |
|  | NX023 | 15 |
|  | NX027 | 15 |
|  | NX029 | 17 |
|  | NX037 | 15 |
|  | NX040 | 40 |
|  | NX041 | 512 |
|  | NX042 | 239 |
|  | NX043 | 1036 |
|  | NX044 | 534 |
|  | NX045 | 749 |
|  | NX046 | 154 |
|  | NX047 | 436 |
|  | NX048 | 898 |
|  | NX049 | 713 |
|  | NX050 | 273 |
|  | NX051 | 621 |
|  | NX052 | 357 |
|  | NX053 | 463 |
|  | NX054 | 363 |
|  | NX055 | 657 |
|  | NX057 | 477 |
|  | NX058 | 270 |
|  | NX059 | 277 |
|  | NX060 | 436 |
|  | NX063 | 346 |
|  | NX064 | 1073 |
|  | NX065 | 209 |
|  | NX066 | 757 |
|  | NX067 | 1077 |
|  | NX069 | 757 |
|  | NX071 | 196 |
|  | NX073 | 228 |
|  | NX075 | 657 |
|  | NX077 | 428 |
|  | NX079 | 613 |
|  | NX081 | 367 |
|  | NX082 | 743 |
|  | NX084 | 24 |
|  | NX087 | 398 |
|  | NX088 | 705 |
|  | NX089 | 795 |
|  | NX094 | 57 |
|  | NX096 | 24 |
|  | NX098 | 1355 |
|  | NX101 | 180 |
|  | NX104 | 225 |
|  | NX105 | 42 |
|  | NX106 | 775 |
|  | NX108 | 521 |
| ZeNix | ZX001 | 17 |
|  | ZX002 | 49 |
|  | ZX003 | 40 |
|  | ZX005 | 31 |
|  | ZX006 | 97 |
|  | ZX009 | 27 |
|  | ZX010 | 18 |
|  | ZX012 | 15 |
|  | ZX013 | 27 |
|  | ZX018 | 15 |
|  | ZX021 | 19 |
|  | ZX024 | 20 |
|  | ZX026 | 21 |
|  | ZX027 | 20 |
|  | ZX028 | 18 |
|  | ZX031 | 24 |
|  | ZX039 | 76 |
|  | ZX040 | 82 |
|  | ZX043 | 48 |
|  | ZX044 | 25 |
|  | ZX047 | 31 |
|  | ZX049 | 108 |
|  | ZX051 | 54 |
|  | ZX052 | 70 |
|  | ZX055 | 65 |
|  | ZX059 | 78 |
