# Supplementary material for: Baseline and acquired resistance to bedaquiline, linezolid and pretomanid, and impact on treatment outcomes in four tuberculosis clinical trials containing pretomanid
Source: PLOS Glob Public Health. 2023 Oct 18;3(10):e0002283. doi: 10.1371/journal.pgph.0002283 (PMC10584172; doi:10.1371/journal.pgph.0002283)
Supplement: S5 Table — (DOCX) [file pgph.0002283.s007.docx]

**S5 Table: Genes completely or partially deleted in pretomanid-resistant isolates from participants ZX026 and/or ST455.**

| **Gene** | **Rv number** | **Product^1^** |
| --- | --- | --- |
| *fadE26** | Rv3504 | Probable acyl-CoA dehydrogenase FadE26 |
| *fadE27* | Rv3505 | Probable acyl-CoA dehydrogenase FadE27 |
| *fadD17* | Rv3506 | Fatty-acid-CoA synthetase FadD17 (fatty-acid-CoA synthase) (fatty-acid-CoA ligase) |
| *PE_PGRS53* | Rv3507 | PE-PGRS family protein PE_PGRS53 |
| *PE_PGRS54* | Rv3508 | PE-PGRS family protein PE_PGRS54 |
| *ilvX* | Rv3509c | Probable acetohydroxyacid synthase IlvX (acetolactate synthase) |
| *Rv3510c* | Rv3510c | Conserved protein |
| *Rv3511* | PE_PGRS55 | PE-PGRS family protein PE_PGRS55 |
| *PE_PGRS56* | Rv3512 | PE-PGRS family protein PE_PGRS56 |
| *fadD18* | Rv3513c | Probable fatty-acid-CoA ligase FadD18 (fragment) (fatty-acid-CoA synthetase) (fatty-acid-CoA synthase) |
| *PE_PGRS57* | Rv3514 | PE-PGRS family protein PE_PGRS57 |
| *fadD19* | Rv3515c | Fatty-acid-CoA ligase FadD19 (fatty-acid-CoA synthetase) (fatty-acid-CoA synthase) |
| *echA19* | Rv3516 | Possible enoyl-CoA hydratase EchA19 (enoyl hydrase) (unsaturated acyl-CoA hydratase) (crotonase) |
| *Rv3517* | Rv3517 | Conserved hypothetical protein |
| *cyp142* | Rv3518c | Probable cytochrome P450 monooxygenase 142 Cyp142 |
| *Rv3519* | Rv3519 | Unknown protein |
| *MTB000157* | ncRv3520 | Putative small regulatory RNA |
| *Rv3520c* | Rv3520c | Possible coenzyme F420-dependent oxidoreductase |
| *Rv3521* | Rv3521 | Conserved hypothetical protein |
| *ltp4* | Rv3522 | Possible lipid transfer protein or keto acyl-CoA thiolase Ltp4 |
| *ltp3* | Rv3523 | Probable lipid carrier protein or keto acyl-CoA thiolase Ltp3 |
| *Rv3524* | Rv3524 | Probable conserved membrane protein |
| *Rv3525c* | Rv3525c | Possible siderophore-binding protein |
| *kshA* | Rv3526 | Oxygenase component of 3-ketosteroid-9-alpha-hydroxylase KshA |
| *Rv3527* | Rv3527 | Hypothetical protein |
| *Rv3528c* | Rv3528c | Unknown protein |
| *Rv3529c* | Rv3529c | Conserved hypothetical protein |
| *Rv3530c* | Rv3530c | Possible oxidoreductase |
| *Rv3531c* | Rv3531c | Hypothetical protein |
| *PPE61* | Rv3532 | PPE family protein PPE61 |
| *PPE62* | Rv3533c | PPE family protein PPE62 |
| *hsaF* | Rv3534c | Probable 4-hydroxy-2-oxovalerate aldolase (HOA) |
| *hsaG* | Rv3535c | Probable acetaldehyde dehydrogenase (acetaldehyde dehydrogenase [acetylating]) |
| *hsaE* | Rv3536c | Probable hydratase |
| *kstD* | Rv3537 | Probable dehydrogenase |
| *Rv3538* | Rv3538 | Probable dehydrogenase. Possible 2-enoyl acyl-CoA hydratase |
| *PPE63* | Rv3539 | PPE family protein PPE63 |
| *ltp2* | Rv3540c | Probable lipid transfer protein or keto acyl-CoA thiolase Ltp2 |
| *Rv3541c* | Rv3541c | Conserved protein |
| *Rv3542c* | Rv3542c | Conserved hypothetical protein |
| *fadE29* | Rv3543c | Probable acyl-CoA dehydrogenase FadE29 |
| *fadE28* | Rv3544c | Probable acyl-CoA dehydrogenase FadE28 |
| *cyp125* | Rv3545c | Probable cytochrome P450 125 Cyp125 |
| *fadA5* | Rv3546 | Probable acetyl-CoA acetyltransferase FadA5 (acetoacetyl-CoA thiolase) |
| ***ddn*** | **Rv3547** | **Deazaflavin-dependent nitroreductase Ddn** |
| *Rv3548c* | Rv3548c | Probable short-chain type dehydrogenase/reductase |
| *Rv3549c* | Rv3549c | Probable short-chain type dehydrogenase/reductase |
| *echA20* | Rv3550 | Probable enoyl-CoA hydratase EchA20 (enoyl hydrase) (unsaturated acyl-CoA hydratase) (crotonase) |
| *Rv3551* | Rv3551 | Possible CoA-transferase (alpha subunit) |
| *Rv3552* | Rv3552 | Possible CoA-transferase (beta subunit) |
| *Rv3553* | Rv3553 | Possible oxidoreductase |
| *fdxB* | Rv3554 | Possible electron transfer protein FdxB |
| *Rv3555c* | Rv3555c | Conserved hypotheticals |
| *fadA6* | Rv3556c | Probable acetyl-CoA acetyltransferase FadA6 (acetoacetyl-CoA thiolase) |
| *Rv3557c* | Rv3557c | Transcriptional regulatory protein (probably TetR-family) |
| *PPE64* | Rv3558 | PPE family protein PPE64 |
| *Rv3559c* | Rv3559c | Probable oxidoreductase |
| *fadE30* | Rv3560c | Probable acyl-CoA dehydrogenase FadE30 |
| *fadD3* | Rv3561 | Probable fatty-acid-CoA ligase FadD3 (fatty-acid-CoA synthetase) (fatty-acid-CoA synthase) |
| *fadE31* | Rv3562 | Probable acyl-CoA dehydrogenase FadE31 |
| *fadE32* | Rv3563 | Probable acyl-CoA dehydrogenase FadE32 |
| *fadE33* | Rv3564 | Probable acyl-CoA dehydrogenase FadE33 |
| *aspB* | Rv3565 | Possible aspartate aminotransferase AspB (transaminase A) (ASPAT) (glutamic--oxaloacetic transaminase) (glutamic--aspartic transaminase) |
| *nat* | Rv3566c | Arylamine N-acetyltransferase Nat (arylamine acetylase) |
| *Rv3566A* | Rv3566A | Hypothetical protein |
| *hsaB* | Rv3567c | Possible oxidoreductase. Possible 3-hydroxy-9,10-seconandrost-1,3,5(10)-triene-9,17-dione hydroxylase |
| *hsaC* | Rv3568c | 3,4-DHSA dioxygenase |
| *hsaD* | Rv3569c | 4,9-DHSA hydrolase |
| *hsaA** | Rv3570c | Possible oxidoreductase. Possible 3-hydroxy-9,10-seconandrost-1,3,5(10)-triene-9,17-dione hydroxylase |
| *kshB* | Rv3571 | Reductase component of 3-ketosteroid-9-alpha-hydroxylase KshB |
| *Rv3572* | Rv3572 | Unknown protein |
| *fadE34* | Rv3573c | Probable acyl-CoA dehydrogenase FadE34 |
| *kstR* | Rv3574 | Transcriptional regulatory protein KstR (probably TetR-family) |
| *Rv3575c* | Rv3575c | Transcriptional regulatory protein (probably LacI-family) |
| *lppH* | Rv3576 | Possible conserved lipoprotein LppH |
| *Rv3577** | Rv3577 | Conserved hypothetical protein |

^1^Annotation as per <https://mycobrowser.epfl.ch/>

*ddn* appears in bold.

Underlined genes were deleted in both isolates.

Asterisk denotes partial deletions.

Shaded genes have been implicated in cholesterol catabolism and/or growth in vivo (S1 Text references [14-17]).
