# Supplementary material for: Baseline and acquired resistance to bedaquiline, linezolid and pretomanid, and impact on treatment outcomes in four tuberculosis clinical trials containing pretomanid
Source: PLOS Glob Public Health. 2023 Oct 18;3(10):e0002283. doi: 10.1371/journal.pgph.0002283 (PMC10584172; doi:10.1371/journal.pgph.0002283)

**S1 Fig.** **Pretomanid REMA MIC distribution for all reported STAND baseline isolates (n=209; blue bars) and the corresponding H37Rv control included with each test run (n=57; orange bars).**


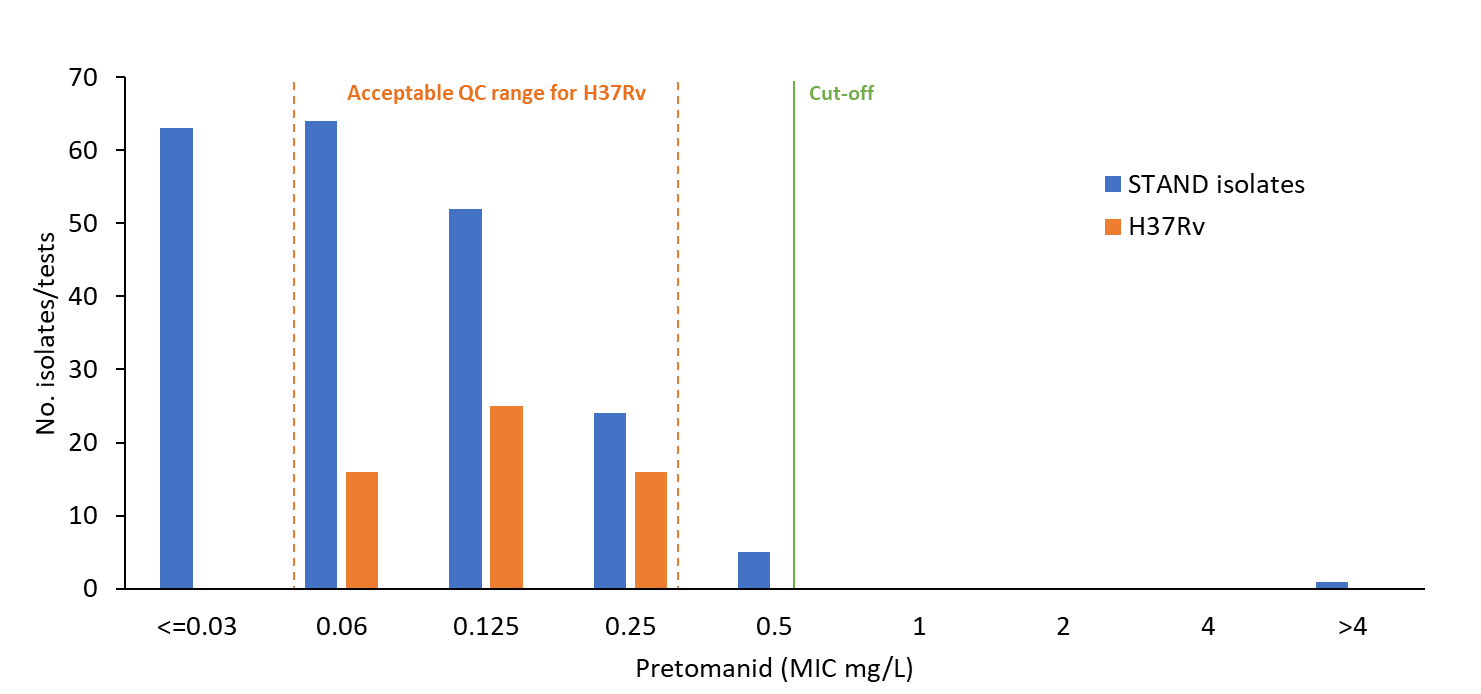

Supplement: S1 Fig — The proposed cutoff for determining resistant isolates based on this data (0.5 mg/L) is indicated by the green line. (DOCX) [file pgph.0002283.s008.docx]
