# Supplementary figures and images for: Baseline and acquired resistance to bedaquiline, linezolid and pretomanid, and impact on treatment outcomes in four tuberculosis clinical trials containing pretomanid
Source: PLOS Glob Public Health. 2023 Oct 18;3(10):e0002283. doi: 10.1371/journal.pgph.0002283 (PMC10584172; doi:10.1371/journal.pgph.0002283)

**S2 Fig. Phylogenetic trees.**

**(A)**


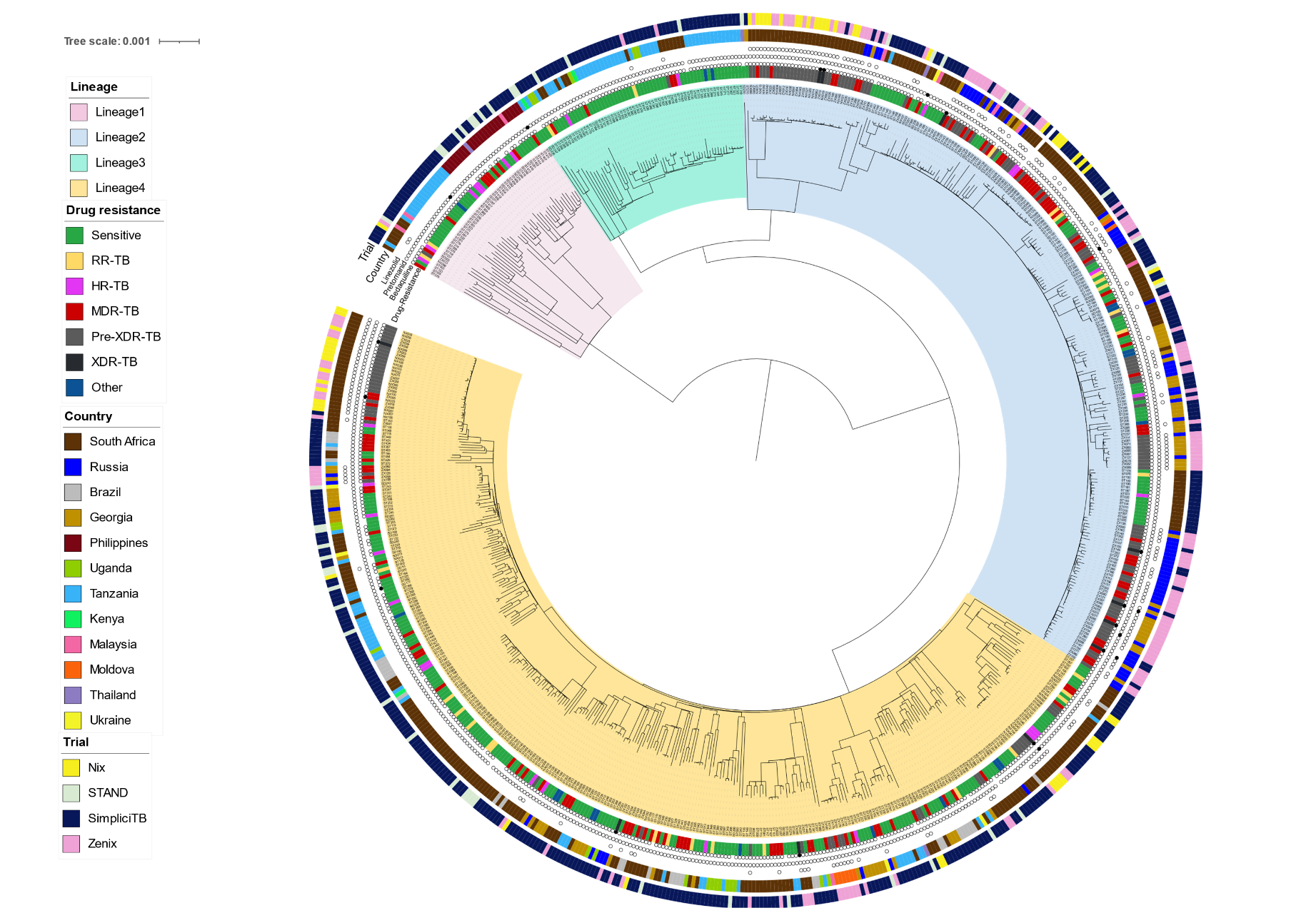


**(B)**


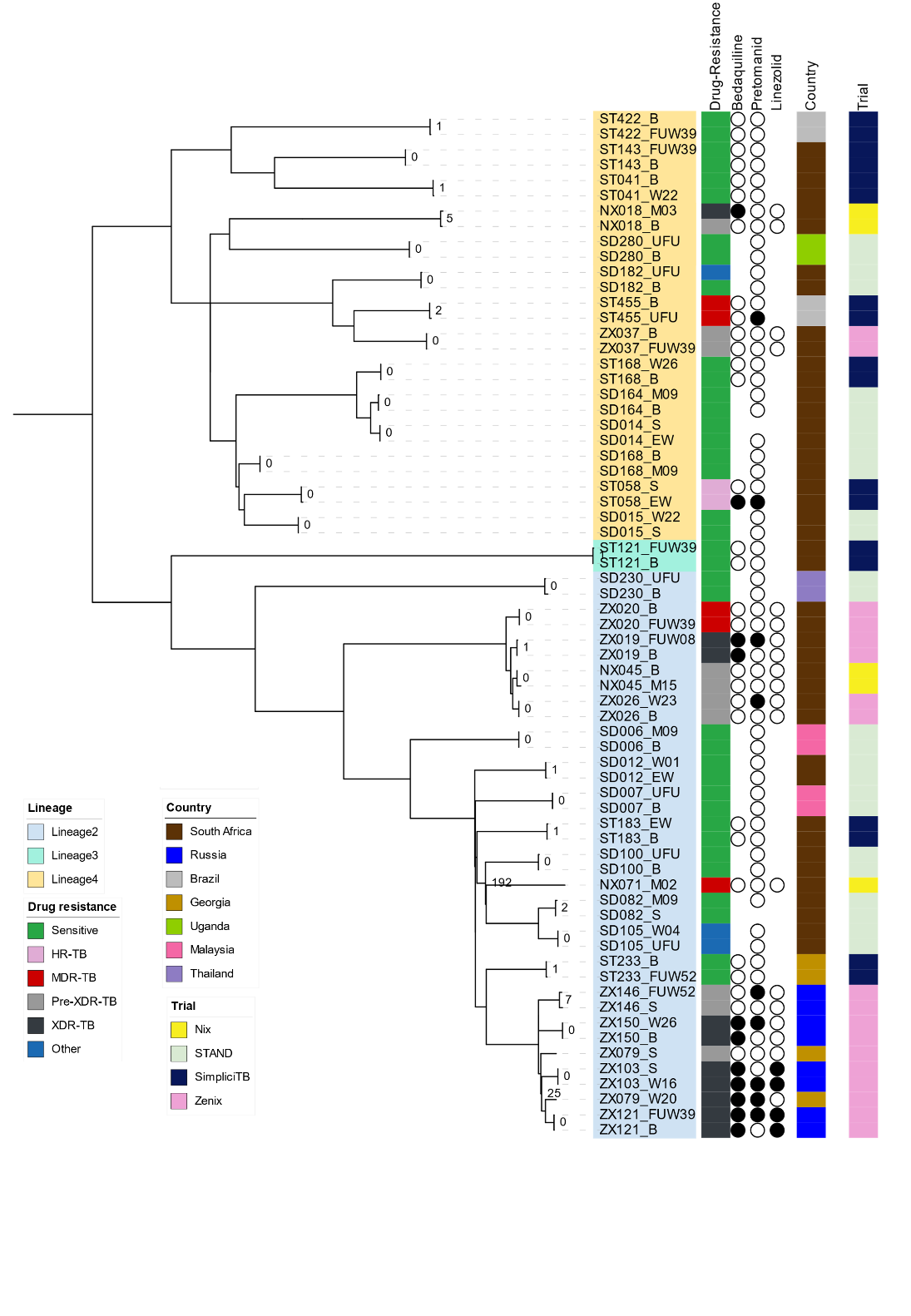

Supplement: S2 Fig — Phylogenetic tree of (A) baseline isolates from all 4 clinical trials for which WGS data available, with lineage shown by the shaded section of the tree. Seven SimpliciTB isolates were excluded from the tree: 6 mixed lineages (ST240, ST336, ST364, ST372, ST400; ZX173) and one Mycobacterium bovis (ST200). Phylogenetic tree of (B) paired baseline and postbaselines isolates from all participants for which WGS available (n = 34). One participant (NX071) has no corresponding baseline. Numbers on the nodes show SNP differences between isolates. Heat maps depict trial, country of clinical trial site, and drug resistance according to WHO 2021 definitions. Phenotypic resistance to pretomanid, bedaquiline and linezolid by MIC testing is shown as circles (open circle: susceptible; closed circle resistant [based of critical concentrations of 2, 1 and 1mg/L for MGIT MIC respectively, and 0.5mg/L for pretomanid REMA MIC]). Visit abbreviations: S—screening; B—baseline; W–week; M–month; FU–follow up; U–unscheduled; EW–early withdrawal. (DOCX) [file pgph.0002283.s009.docx]
