# Supplementary material for: Baseline and acquired resistance to bedaquiline, linezolid and pretomanid, and impact on treatment outcomes in four tuberculosis clinical trials containing pretomanid
Source: PLOS Glob Public Health. 2023 Oct 18;3(10):e0002283. doi: 10.1371/journal.pgph.0002283 (PMC10584172; doi:10.1371/journal.pgph.0002283)

**S3 Fig. Summary data for the 10 participants that acquired resistance to pretomanid and/or bedaquiline.**

1. **NX018**

**
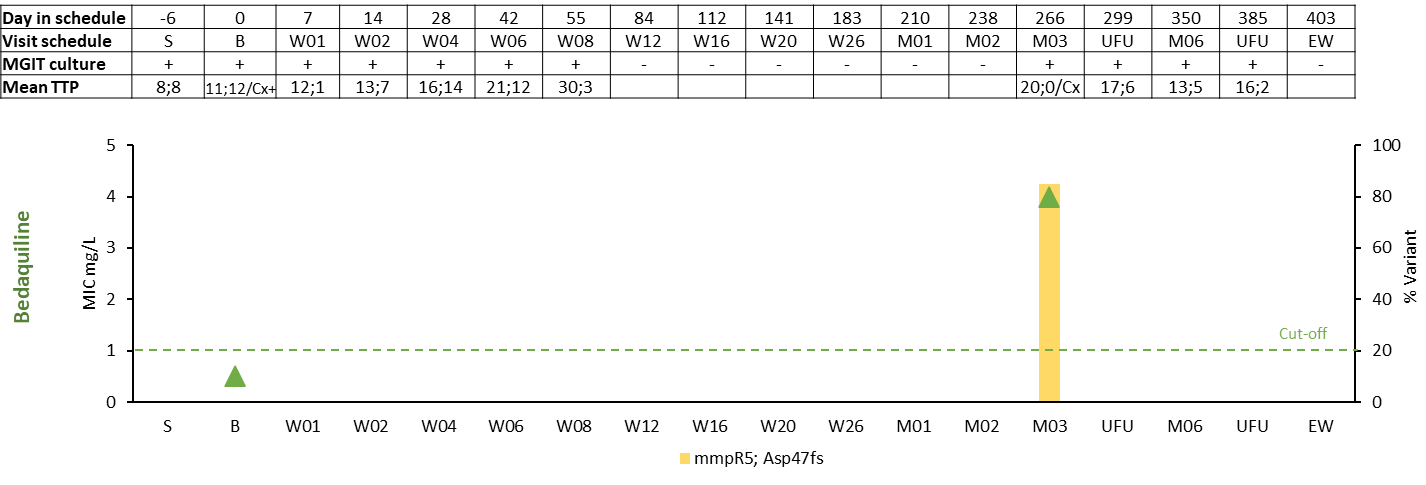
**

**(B) ZX019**

**_
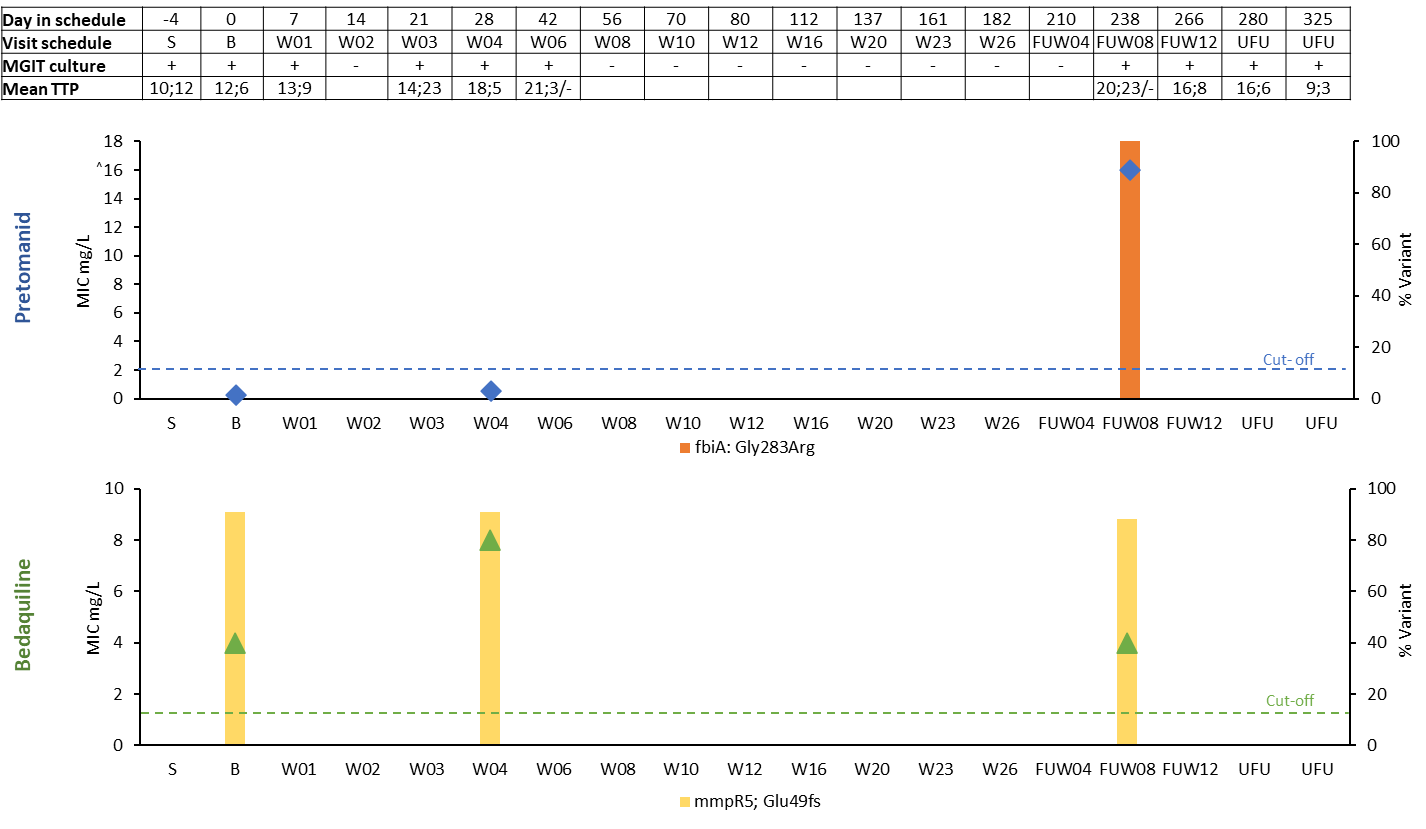
_**

**(C) ZX026**

**
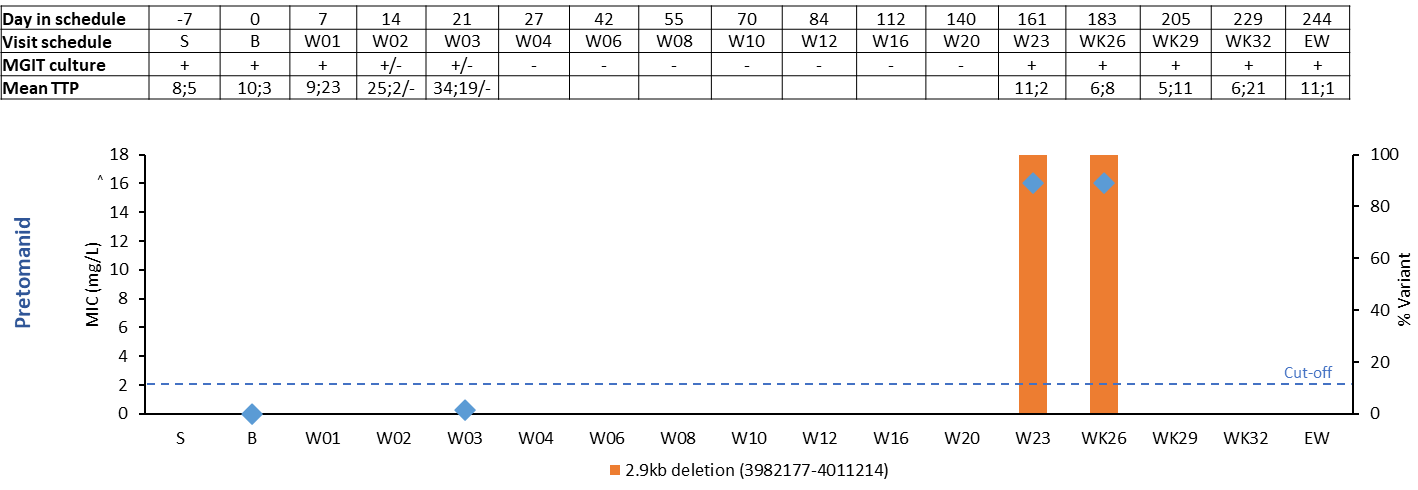
**

**(D) ZX079
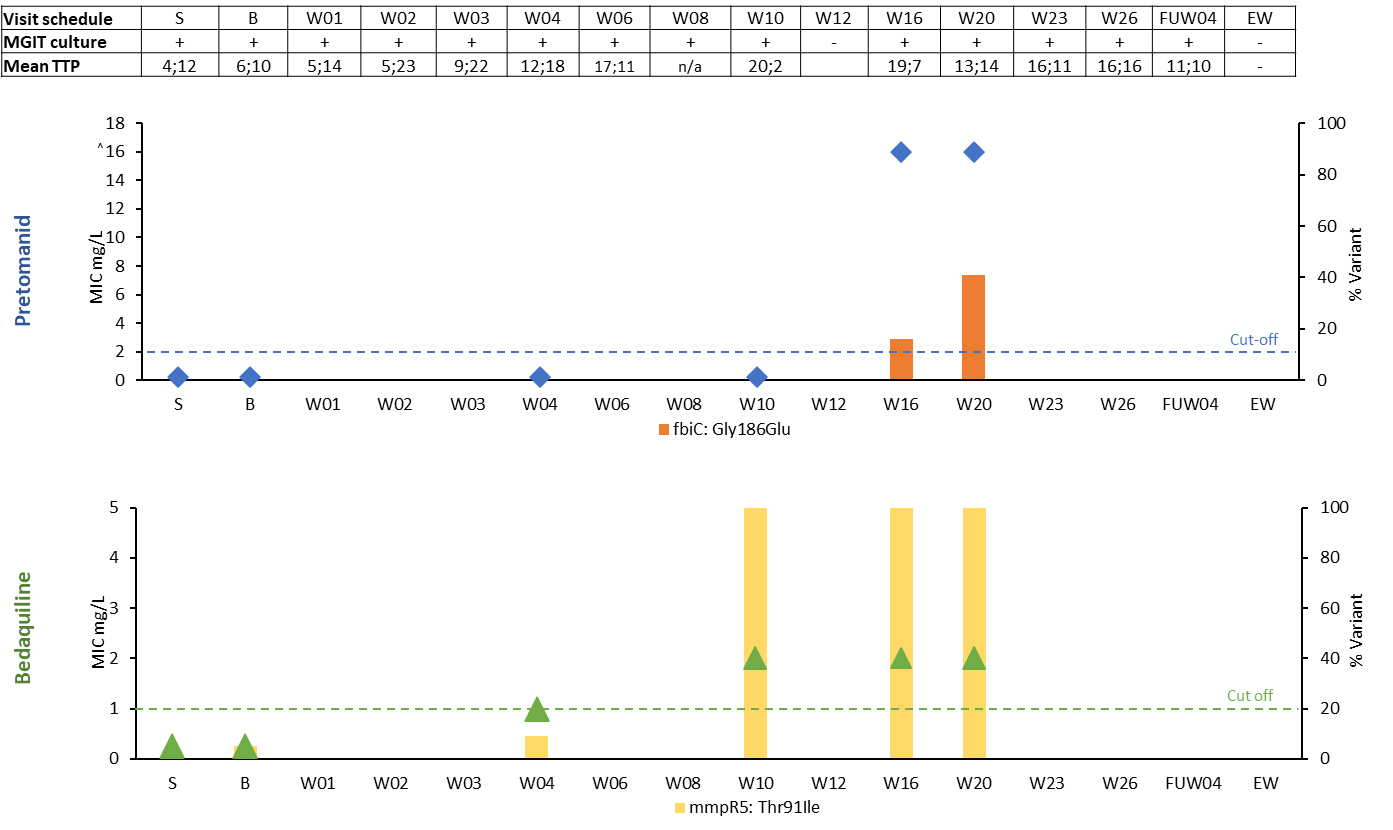
**

**
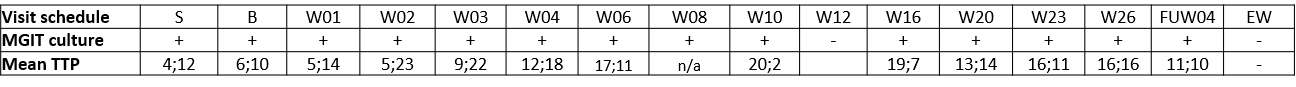
**


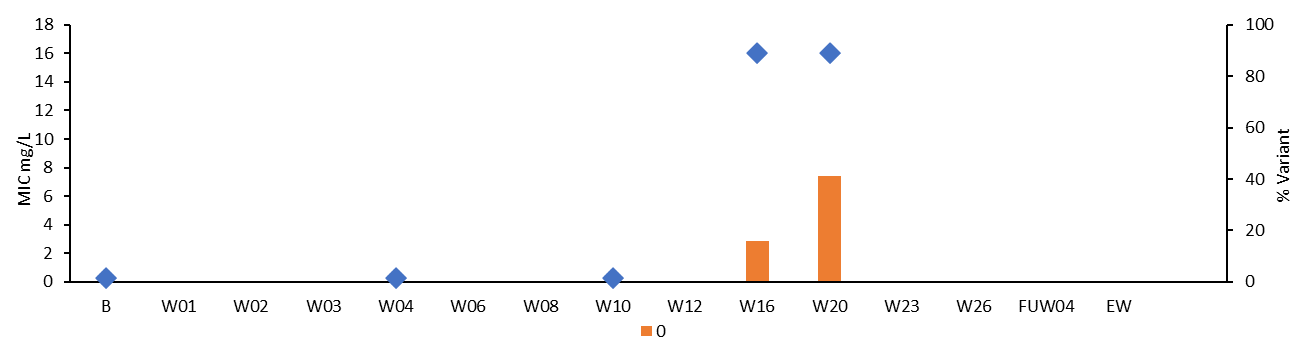


**Pretomanid**

Cut-off

^

**Bedaquiline**

Cut off

**(E) ZX103**

**
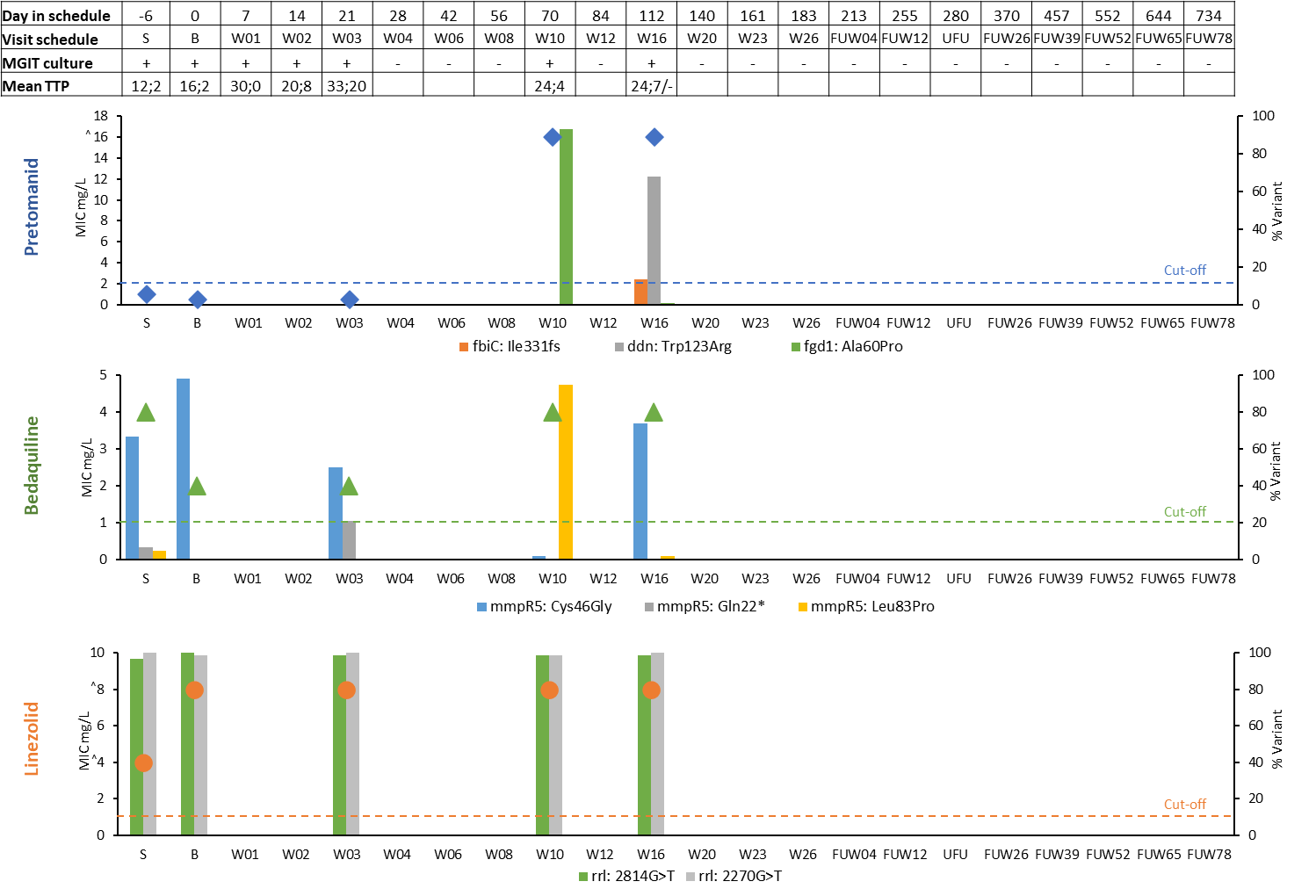
**

**(F) ZX121**

**
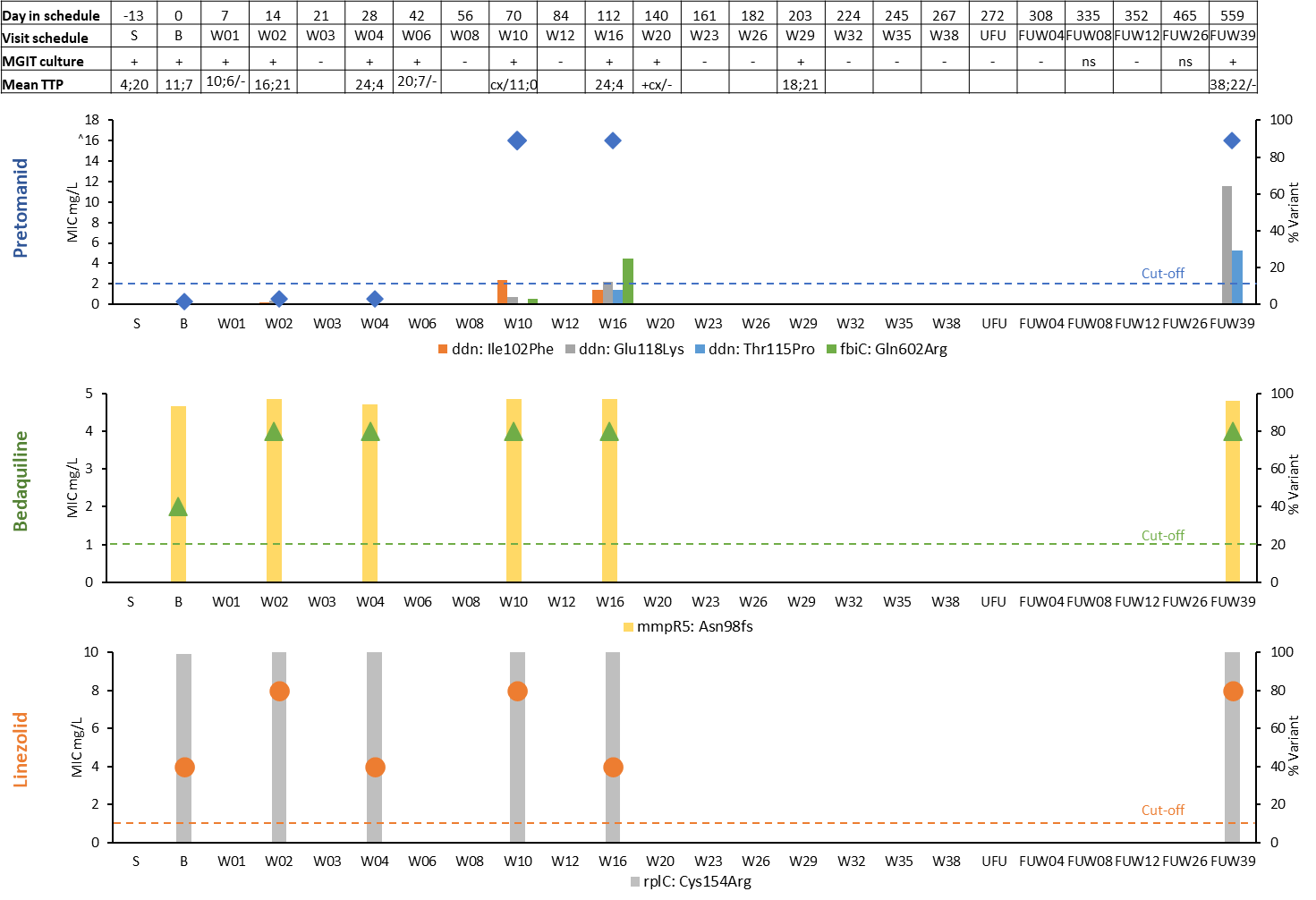
**

**(G) ZX146**

**_
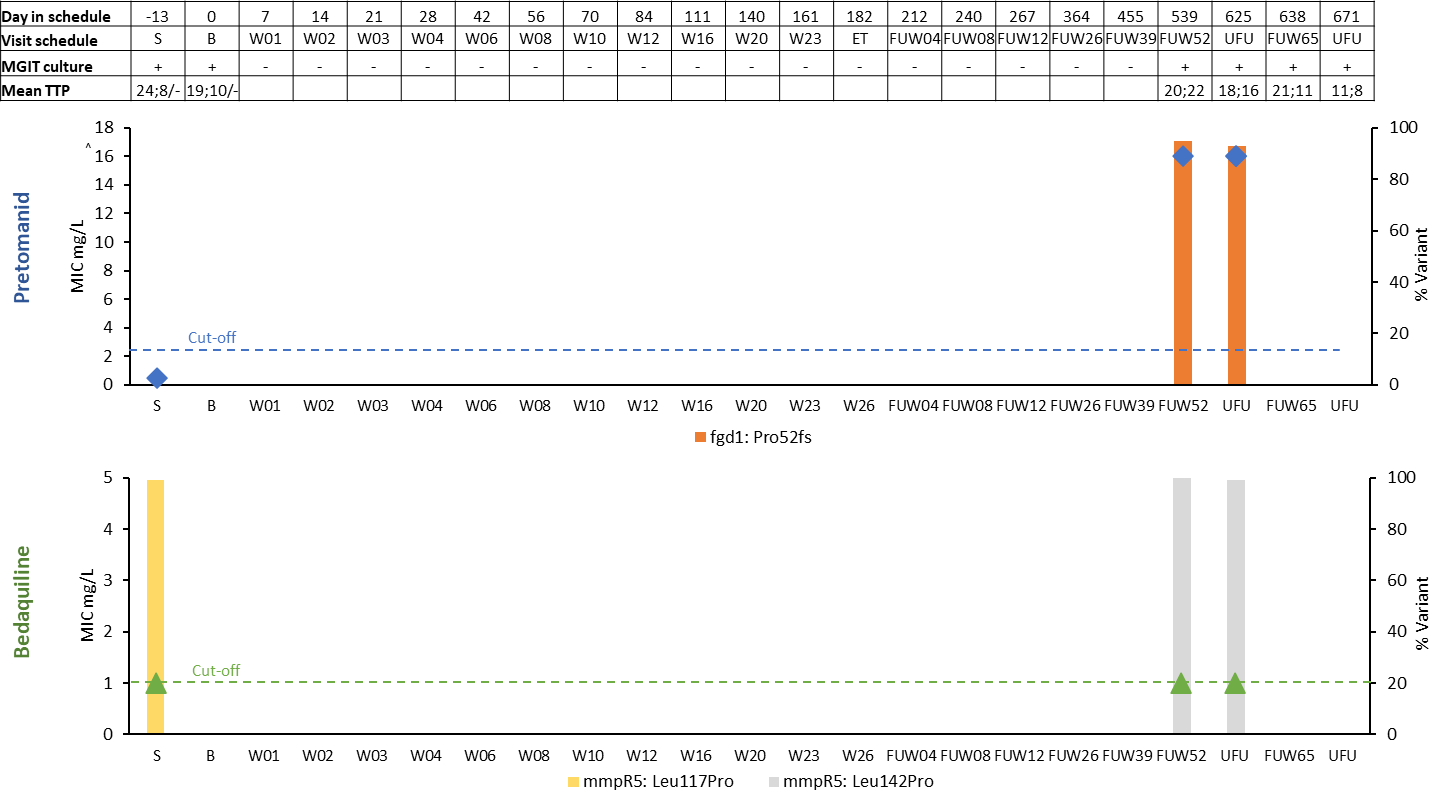
_**

**NOTE:** ZX146 has a bedaquiline MIC of 1mg/L (at the cut-off but not considered resistant). Bedaquiline MIC and variant data included here as variants in *mmpR5* were detected.

**(H) ZX150**

**_
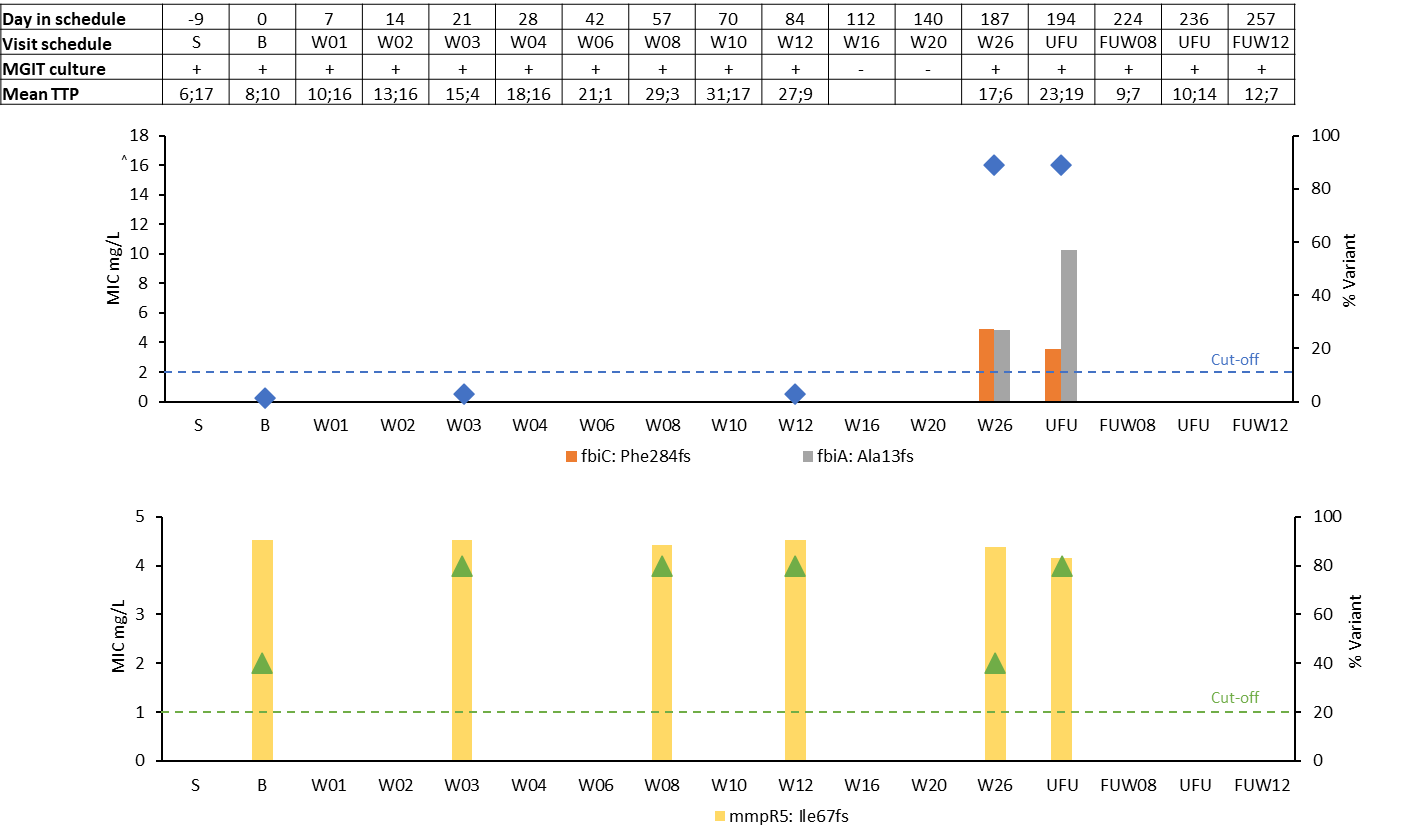
_**

**(I) ST058**

**
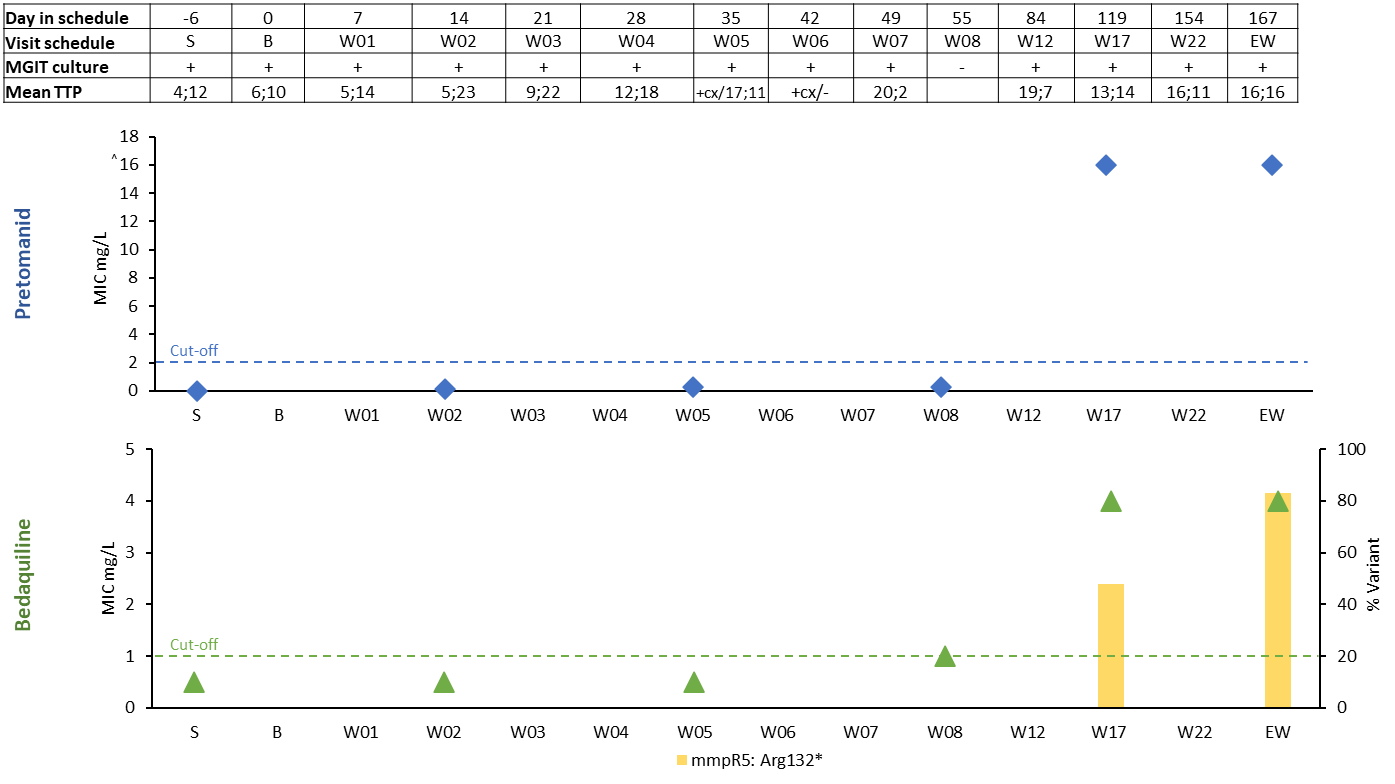
**

**(J) ST455**


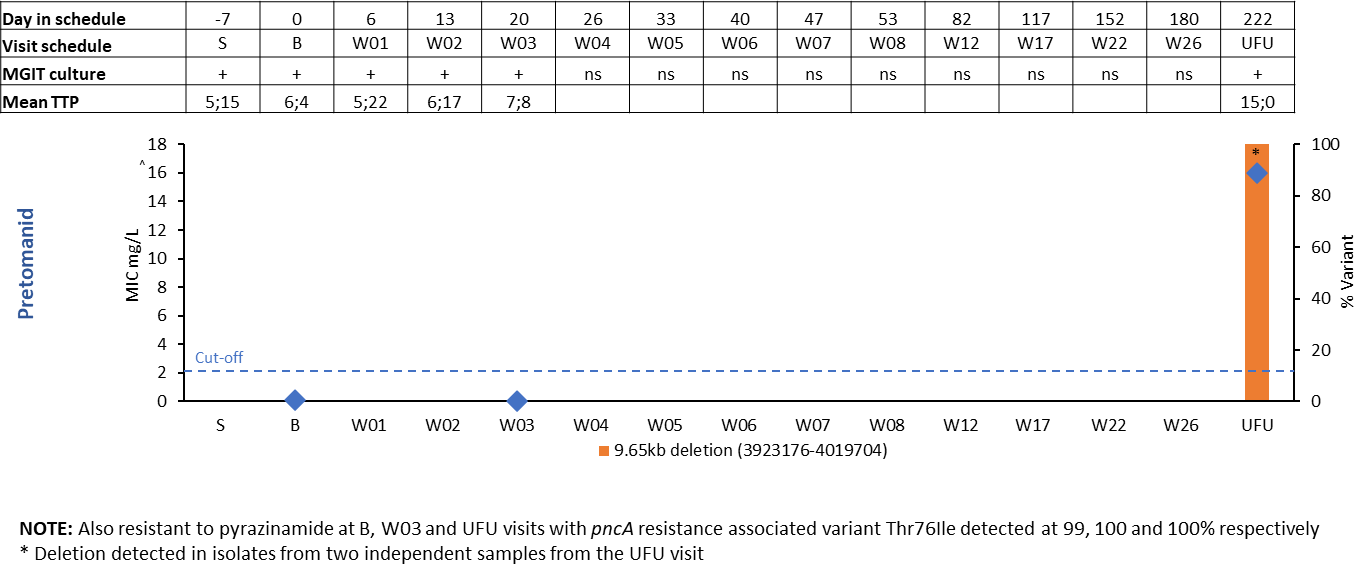

Supplement: S3 Fig — Tables show the visit schedule, and the corresponding day in the visit schedule relative to the baseline visit as day 0 (S: screening; B: baseline, W: week; M: month, UFU: unscheduled visit in follow up period; EW: early withdrawal). Primary MGIT culture results from the two sputum samples collected at each visit are shown as an overall status from the visit (MGIT culture +/-), where any positive MGIT results in positive status; and as a mean time to positivity (TTP), represented as days; hours, where two positive MGIT cultures available, or otherwise as the TTP of the single positive MGIT culture (Cx: contaminated/AFB-; Cx+: Contaminated/AFB+/MTB confirmed; ns: no sputum produced). Graphs show MIC data for pretomanid (blue diamond), bedaquiline (green triangle) and linezolid (orange circle) over time, and bars representing % of variants in the resistance genes of interest (see Table 5 and S3 Table) from the WGS data. Dashed lines define the cut-off for resistance. ^ MIC concentration was the highest tested and MIC is greater than the value shown. (DOCX) [file pgph.0002283.s010.docx]
