# Supplementary material for: Baseline and acquired resistance to bedaquiline, linezolid and pretomanid, and impact on treatment outcomes in four tuberculosis clinical trials containing pretomanid
Source: PLOS Glob Public Health. 2023 Oct 18;3(10):e0002283. doi: 10.1371/journal.pgph.0002283 (PMC10584172; doi:10.1371/journal.pgph.0002283)
Supplement: S4 Fig — Distributions of plasma concentrations for study drugs in (A) Nix-TB; (B) ZeNix and (C) SimpliciTB for all participants who acquired resistance to pretomanid and/or bedaquiline. Each plot shows the overall distribution of observed concentrations as boxplots, on which are superimposed individual observations for the participants of interest. Pre- and post-dose concentrations were observed at various visits as indicated. Only pre-dose concentrations were observed in Nix-TB (A). (DOCX) [file pgph.0002283.s011.docx]

**S4 Fig. Distributions of plasma concentrations for study drugs in (A) Nix-TB; (B) ZeNix and (C) SimpliciTB for all participants who acquired resistance to pretomanid and/or bedaquiline.**


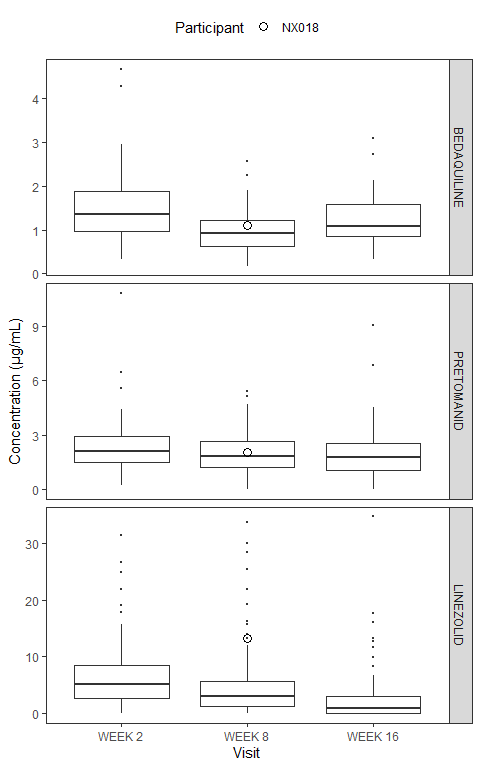


**(A)**


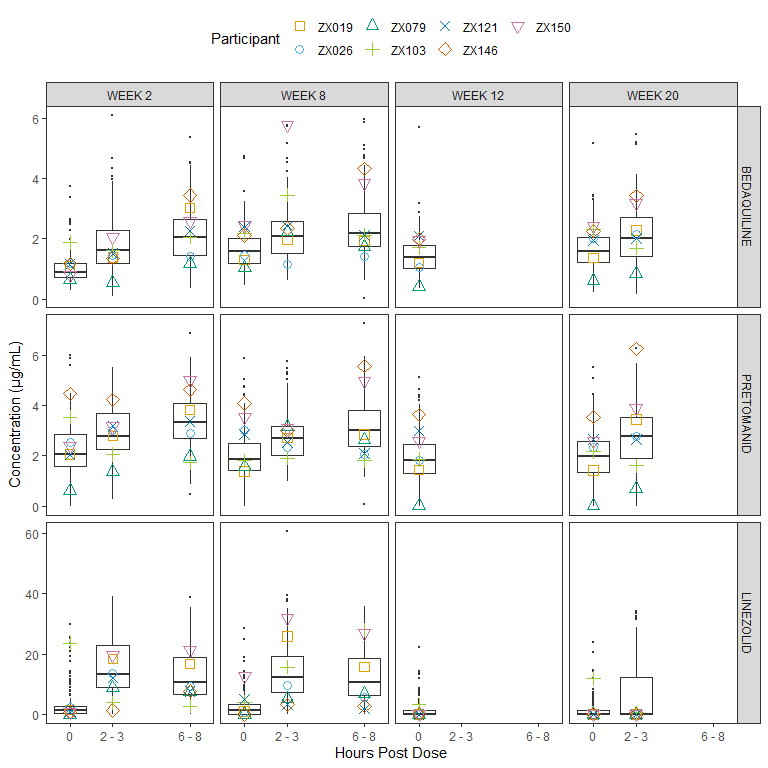


**(B)**


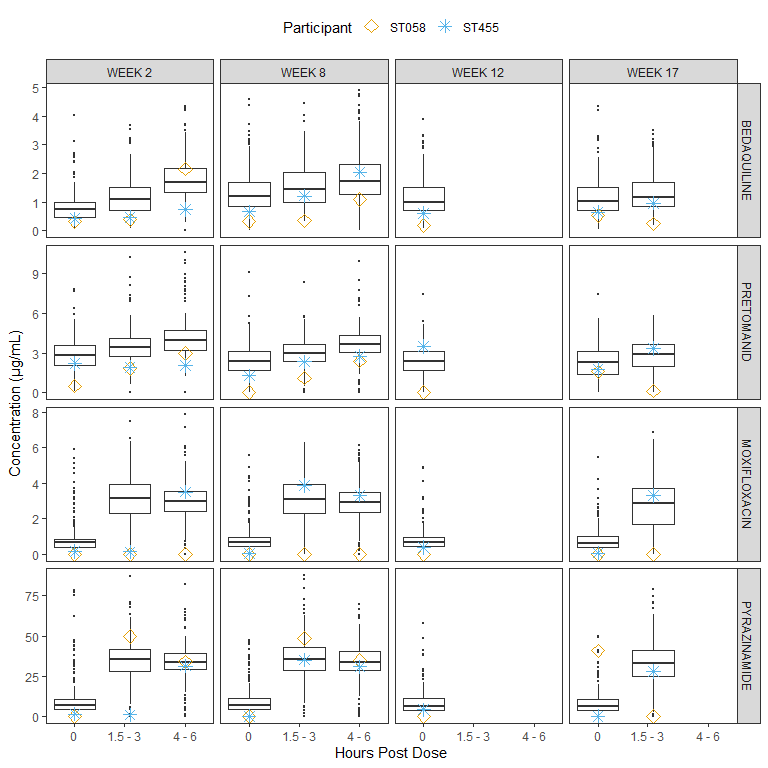


**(C)**
