# Supplementary material for: Baseline and acquired resistance to bedaquiline, linezolid and pretomanid, and impact on treatment outcomes in four tuberculosis clinical trials containing pretomanid
Source: PLOS Glob Public Health. 2023 Oct 18;3(10):e0002283. doi: 10.1371/journal.pgph.0002283 (PMC10584172; doi:10.1371/journal.pgph.0002283)
Supplement: Attachment — Submitted filename: Responses to Reviewers.docx [file pgph.0002283.s012.docx]

Dear Dr. Muñiz-Salazar,

We thank you and the Reviewers for your careful review of our manuscript. Please find below our responses to each point raised by the Reviewers.

We also want to make you aware of some, more editorial, changes to the manuscript:

- We added a new author, Dr. Morounfolu Olugbosi (a PDF of emails showing that all other authors are in agreement with this decision has now been submitted).
- In addition to changes suggested by the Reviewers, we made some minor corrections throughout the main manuscript (“MainBody”), as well as S1 Text, S3 Figure and S1 Appendix.
- Regarding the Appendix, we have been recently advised not to include participants’ full Date of Randomization (we kept only the year) as this could be used to identify the participants. The same holds true for Date of Sputum Collection, which has now been removed. The approximate timing of sputum collection (Column ‘Visit’) which is really needed for understanding the analyses, remain. On the other hand, we have now added the ENA numbers corresponding to all WGS data to the file.
- We have not yet reorganized the references in this new version; we will wait for approval of the final text to do so. Having said this, only one reference was removed, and a new added.

Again, thank you for your time and expertise.

Best regards,

Juliano Timm

**Rebuttal**

Reviewer #1

| **Reviewer’s comment** | **Authors’ response** |
| --- | --- |
| 1. An introduction about the global burden of TB should be provided in the beginning of the introduction. Adding a few sentences on what has been the shortcomings of the regimen prior to BPaL (INH, rifampicin) should be provided to then pave way for the need to include BPaL – based regimens. | We have now included the global burden of TB and provided more context for the introduction of BPaL. Please note that BPaL/M is currently not recommended for the treatment of drug-sensitive TB, ie., not meant to replace the standard of care (SoC) based on INH and rifampicin. A discussion on the shortcomings of the SoC is beyond the scope of this manuscript. |
| 2. Line 44 – which specific populations/ epidemiological regions has WHO enlisted in the new regimens should be mentioned for readers not familiar with the details. | This point is clarified in the revised first paragraph. |
| 3. Line 45 states “in clinical trials” but only cites a single reference at the end of the sentence. | Please see the added sentence. The reference provided is the link to a website maintained by Stop TB Partnership which describes all TB drug trials underway, including those for which publications are not available yet. |
| 4. Line 47 – please mention which combination therapies it was approved as part of. | Please note that neither FDA nor EMA labels for bedaquiline specify a regimen. The drug was originally approved as an “add-on” drug. Nevertheless, the most relevant recommendation is the one from WHO – for use in all-oral regimens for MDR/RR-TB, detailed in REF #2. |
| 5. Lines 50-53 – please provide adequate background information on published literature on Mtb drug resistance before introducing the biochemistry details. | We reorganized the introduction and moved the text about what’s known in terms of prevalence of resistance to the BPaL drugs (now second paragraph) before going into the biochemical/genetic details for each drug. |
| 6. Line 53 – No reference. | REF #7 added here. |
| 7. Line 55 – What type of Mtb cells? No reference. | We made it clearer we are referring to the bacterial cells by using the term “tubercle bacilli”. Also added REF #6 here. |
| 8. Line 56 – What is the rational of mentioning clofazimine? | This mechanism of resistance to bedaquiline – overexpression of the MmpL5/MmpS5 pump – also confers resistance to clofazimine, another TB drug. In other words, there is cross-resistance between the two drugs. |
| 9. Overall, the introduction needs to be improved in making the connecting dots in between change of paragraphs. In its current format, each paragraph is a stand-alone entity and fails to provide a connected picture of the study. | As mentioned above, we reorganized the introduction and, hopefully, better connected the different paragraphs. |
| 10. Line 77 – 81 is too long and confusing to read. | We shortened/modified the sentence (now starting Line 52). |
| 11. Line 83 – The authors should provide a reason and references to substantiate this outcome of measuring only bedaquiline resistance and in small number of countries as compared with the other two drugs? | We believe the Reviewer misunderstood the point; there is even less data/publications on linezolid or delamanid/pretomanid resistance prevalence. We modified the sentence for more clarity (now Line 58).  We now also emphasize that ours is the first large study reporting pretomanid susceptibility data in the clinic. |
| 12. Table 1 – Is it possible to provide the timelines for post-baseline measurements in these cohorts? | General criteria for selection of post-baseline isolates are described in Methods (now Lines 133-137). And the specific timeline for each post baseline isolate analyzed is included in S1 Appendix. This information is too complex to fit in Table 1. |
| 13. Is there a reference that can be added at Line 121? | Instead of providing a reference for each laboratory procedure performed at the Central Laboratory (UCL; same for all trials), we have now decided to provide a link to the Laboratory Manuals that describe all these procedures in detail – please see now Line 130. All readers will be able to access these manuals. |
| 14. Can the authors state how comparable were the time points of sputum collection between the 4 cohorts? | The time points of sputum collection and culture were very similar across the 4 trials. We added a sentence along these lines (Line 136). We also included a new table in S1 Text that shows the minor schedule differences between the trials. |
| 15. Lines 127 – 129: Please provide references for the protocols used. | Please see response to comment #13 |
| 16. How do the authors factor in the variation (if any) from the different MIC assays used in the cohorts? How do they factor that user variations when retesting the samples? What controls were used? | MIC assays were performed in the same laboratory (UCL) following the same SOP, during the 4 trials. As described on 2^nd^ paragraph, page 9, H37Rv control strain was used as a control in each batch of MIC testing. Batches with a H37Rv result outside of predetermined QC ranges were considered invalid and repeated. |
| 17. Why were only 694 isolates genotyped out of a total of 859 baseline Mtb isolates? Was there any particular cohort that had the majority of baseline WGS data missing? Does this present a confounding factor in this type of comparative analysis? | As shown in Table 1, only 48 baseline isolates from STAND were sequenced. That’s because the STAND Protocol did not mandate baseline sequencing; this was only implemented in the other, more recent trials. Nevertheless, this should not be seen as a confounding factor. WGS was primarily performed to determine the genetic mechanisms of resistances previously detected by phenotypic DST/MIC. For STAND, 212/284 (75%) of baseline isolates could be tested by phenotypic DST/MIC and, among these, the only baseline isolate with resistance to pretomanid was sequenced (Table 5). |
| 18. Line 83: Did the authors look deeper into other factors to see why this variation in susceptibility to pretomanid existed in different locations? | We are not sure what the reviewer is referring to; Introduction L83 discusses results of a bedaquiline resistance surveillance study. |
| 19. Line 240: How long after treatment completion did the participant relapse? | This participant relapsed 83 days after treatment completion. We have now updated Tables 3 & 4, including the same information for all patients that relapsed. |
| 20. Table 4 lists the participants who acquired resistance with no baseline resistance to bedaquiline and/or linezolid and/ or pretomanid. However, 50% of the participants listed were withdrawn from the treatment due to different reasons. It is not clear what the take home message here really is? | The main purpose of this table is to list all participants with no baseline resistance who acquired resistance during the trials, along with the regimen they received and their clinical outcomes and the reasons for the outcomes (all had unfavorable outcomes). This data is presented in support of the text on Pages 6 & 23 which refers to, e.g., acquired resistance in this group was very rare, the fact that two of these participants had compliance issues. |
| 21. Line 245: It might be a little premature to link bedaquiline resistance as a risk for pretomanid AR unless further evidence exists to link the two mechanistically. | The link between bedaquiline BR and pretomanid AR is not mechanistic. The two drugs have very different resistance mechanisms, as discussed in the Introduction. Bedaquiline BR appeared a risk factor for pretomanid AR in Nix-TB/ZeNix simply because a BPaL-treated patient who has bedaquiline BR is presumably getting a combination of only 2 effective drugs, PaL. Pretomanid would be less protected in this case. |
| 22. Line 258: True but what about longer term relapse? | As mentioned in the legend of Table 3 and now a new table in S1 Text, participants were followed up to 1.5-2 years after treatment completion, depending on the trial. In programmatic conditions, most TB patients relapse within 6 months of follow-up (please see, eg., <https://www.ncbi.nlm.nih.gov/pmc/articles/PMC3354489/>) |
| 23. Could the authors mention the fitness levels of the Mtb isolates from these cohorts and if that had a role to play in the mutation rates observed? | A study of the fitness and the mutation rate of >20 resistant Mtb isolates identified here, *in vitro,* would be interesting but was beyond the scope of this work. |
| 24. Lines 274 – 277: Were these observations made in different cohorts or same cohorts? Does the epidemiology or treatment regimens play a role? | The turnover of multiple alleles was only observed in ZeNix, but this could be simply because most (7/10) participant isolates included in this analysis were from this trial. We agree with the Reviewer that this is something to look at in future studies, the effect of regimen composition on number/pattern of resistance mutations, most likely using “deep sequencing” techniques. |
| 25. How translational are the findings when applied to a larger cohort given the small numbers of resisters and no statistical significance observed? | Please note that a comparison of bedaquiline BR in Nix-TB/ZeNix vs. SimpliciTB was statistically significant (Table 2). Other comparisons did not achieve statistical significance because the numbers of resistant isolates were too low. We believe this “problem” (if one can talk about low resistance rates in a drug trial as a problem) will only be overcome with the analysis of very large cohort of patients treated with BPa-based regimens, which will only become available after a few years of programmatic implementation. Our study includes the largest cohorts of patients treated with pretomanid as of today. |

Reviewer #2

**Major comments:**
An important message from the paper’s main findings should be made more clearly. Resistance rates were rare, even in patients highly resistant to other agents, there was great variation in the underlying mutations, mot all mutations found during in vitro testing were clinically relevant, and in quite a few cases the reason for resistance could not be found at all (T5). This shows how difficult, if not impossible, it would be to have rational methods for resistance testing rolled out at low cost together with novel drugs and regimens. It would not be possible to rationally design such tests without the experience of a few years of usage in the field. This should be added to the discussion prominently.

**Authors’ response:**

We appreciate the Reviewer’s assessment that our paper has important messages. We added a new paragraph (starting L354) discussing those issues.

Minor comments:

| **Reviewer’s comment** | **Authors’ response** |
| --- | --- |
| Abstract | |
| Add percent to every number. | Percentages added to the Abstract, Table 1 and throughout the manuscript, when appropriate. |
| L37: The conclusion about the protection of drugs is valid, however, it must also be considered that there is more than just a drug combination that avoids resistance at play. Clinical trials for drug or regimen registration will ensure that underdosing or poor compliance are avoided. Also, the study population is selected and looked after with much larger resources than available upon implementation in the field (this goes for a few other places where this claim is made). | Point well taken and addressed, not in the Abstract because of the word count limit, but in the Discussion (Line 336).  Please note we use the words “in support of/suggest” in the Abstract. |
| Introduction | |
| L45 and other places: In the context of these novel drugs and regimens I would recommend not to use definitions that might in a few years be obsolete. DS, DR, RR, MDR, pre-XDR etc are concepts based on currently available regimens that will hopefully soon be replaced with better ones. I would recommend, where possible, to name the agents that this refers to, and remain conscious about how this will read to a person in 10- or 20-years’ time. At some point it will no longer be intuitive what is meant by DS-TB and DR-TB. | We agree with the Reviewer that these definitions are sometimes confusing and will probably be obsolete in a few years. However, we feel we must keep them here for the following reasons:   - The trials discussed here were planned and conducted several years ago, when even pre-2021 WHO definitions were widely used. The protocols of these trials used such definitions for participant eligibility purposes; and their results were usually stratified along those definitions, as shown in the corresponding publications. - The labels of both bedaquiline and pretomanid refer to those terms. - WHO recommends the use of these drugs for particular populations along those definitions.   Please note that we carefully explain each definition/term in the manuscript. |
| L69: Consider replacing production with synthesis. | Modified as suggested. |
| L78: Introduce delamanid above under nitroimidazoles. | Delamanid is now introduced in the 1^st^ paragraph of introduction. |
| L81: I disagree that it’s only the limited capacity. There is also our limited knowledge what to look for, which is the very reason the research was done that is reported here. | We modified the sentences (now L55-59) to acknowledge that. |
| L97: instead of 7 years say from xxxx to xxxx | Replaced by “February, 2015 to March, 2020” (not 7 years). |
| Results | |
| T1 and L139: I was a bit confused about if the is 170 (as per table totals 1029 minus 859) or 169 as per text. This won’t make a major difference but please check that this is all correct and clear. | This discrepancy is because Table 1 refers specifically to participants with Pa MIC data. For one participant, it was possible to obtain bedaquiline/linezolid MICs, but not a pretomanid MIC, due to technical difficulties. We have now clarified the text in Methods, Line 171. |
| L125: any participant that still has an isolate at M4 is at high risk of having a poor outcome. Perhaps rewrite what is meant with “regardless of …” | We amended the text for more clarity (now L135). |
| Results: Throughout, also on the tables, present % not only numbers. | Please see response to 1^st^ comment. |
| T2 L204. Perhaps find a more diplomatic way to explain what those authors did, which was also passed by reviewers and editors. There must be a good reason to do it the way they did, even if these authors disagree. | We believe this was an oversight from the authors of that, otherwise excellent, review. We don’t see how 109 (baseline isolates) could be used as the denominator when only data for 58 were available. In any case, we modified the sentence. |
| L206/207: Some of this text can be shortened. | We shortened the sentence starting L211. |
| L213 – how was this history examined? This is not quite in line with the claim that the data was anonymized for analysis. What information was available and how was it accessed? | All the analyses were performed on non-anonymized data; we did not claim otherwise. We only used “dummy” participant IDs throughout the manuscript to avoid exposing their true identities (as we are obliged to do). Along these lines, in the modified S1 Appendix, we have now removed full visit dates (keeping only the year) and sputum collection dates, as they also posed a risk. |
| T3: It is interesting that there was not a single patient without previous TB, i.e., that was de-novo infected with a resistant strain. So no transmission of resistant TB was observed in this cohort. This is a bit in contrast with some claims that transmission of resistant TB is a driver of resistance in the field. Can this somehow be explained with the population selection, or a different theory? | We now realize that the way we presented the “TB history” in Table 3 was misleading, and led the Reviewer to believe that all patients with baseline resistance had a prior history of TB. In fact, those with a very short TB history duration – eg., SD236, ST282, ST335, ST441 & ZX077 – were newly-diagnosed patients. As a matter of fact, in both STAND & SimpliciTB, only newly-diagnosed patients or those with no history of TB treatment within 3 years prior to screening were eligible.  Therefore, some participants in our trials had probably been recently infected with resistant MTB, while others probably acquired these resistances while treated for TB.  We added a footnote to Table 3 to make this clear. |
| Discussion: This is generally a bit lengthy and often repeating what has already been nicely presented. It should be shortened and focused on what the findings mean rather than repeating them. | We shortened some text already present but included additional text to address the Reviewer’s Major Comment. |
